# The effects of small class sizes on students' academic achievement, socioemotional development and well‐being in special education: A systematic review

**DOI:** 10.1002/cl2.1345

**Published:** 2023-07-14

**Authors:** Anja Bondebjerg, Nina Thorup Dalgaard, Trine Filges, Bjørn Christian Arleth Viinholt

**Affiliations:** ^1^ VIVE—The Danish Centre for Social Science Research Copenhagen Denmark

## Abstract

**Background:**

Class size reductions in general education are some of the most researched educational interventions in social science, yet researchers have not reached any final conclusions regarding their effects. While research on the relationship between general education class size and student achievement is plentiful, research on class size in special education is scarce, even though class size issues must be considered particularly important to students with special educational needs. These students compose a highly diverse group in terms of diagnoses, functional levels, and support needs, but they share a common need for special educational accommodations, which often entails additional instructional support in smaller units than what is normally provided in general education. At this point, there is however a lack of clarity as to the effects of special education class sizes on student academic achievement and socioemotional development. Inevitably, such lack of clarity is an obstacle for special educators and policymakers trying to make informed decisions. This highlights the policy relevance of the current systematic review, in which we sought to examine the effects of small class sizes in special education on the academic achievement, socioemotional development, and well‐being of children with special educational needs.

**Objectives:**

The objective of this systematic review was to uncover and synthesise data from studies to assess the impact of small class sizes on the academic achievement, socioemotional development, and well‐being of students with special educational needs. We also aimed to investigate the extent to which the effects differed among subgroups of students. Finally, we planned to perform a qualitative exploration of the experiences of children, teachers, and parents with class size issues in special education.

**Search Methods:**

Relevant studies were identified through electronic searches in bibliographic databases, searches in grey literature resources, searches using Internet search engines, hand‐searches of specific targeted journals, and citation‐tracking. The following bibliographic databases were searched in April 2021: ERIC (EBSCO‐host), Academic Search Premier (EBSCO‐host), EconLit (EBSCO‐host), APA PsycINFO (EBSCO‐host), SocINDEX (EBSCO‐host), International Bibliography of the Social Sciences (ProQuest), Sociological Abstracts (ProQuest), and Web of Science (Clarivate, Science Citation Index Expanded & Social Sciences Citation Index). EBSCO OPEN Dissertations was also searched in April 2021, while the remaining searches for grey literature, hand‐searches in key journals, and citation‐tracking took place between January and May 2022.

**Selection Criteria:**

The intervention in this review was a small special education class size. Eligible quantitative study designs were studies that used a well‐defined control or comparison group, that is, studies where there was a comparison between students in smaller classes and students in larger classes. Children with special educational needs in grades K‐12 (or the equivalent in European countries) in special education were eligible. In addition to exploring the effects of small class sizes in special education from a quantitative perspective, we aimed to gain insight into the lived experiences of children, teachers, and parents with class size issues in special education contexts, as they are presented in the qualitative research literature. The review therefore also included all types of empirical qualitative studies that collected primary data and provided descriptions of main methodological issues such as selection of informants, data collection procedures, and type of data analysis. Eligible qualitative study designs included but were not limited to studies using ethnographic observation or field work formats, or qualitative interview techniques applied to individual or focus group conversations.

**Data Collection and Analysis:**

The literature search yielded a total of 26,141 records which were screened for eligibility based on title and abstract. From these, 262 potentially relevant records were retrieved and screened in full text, resulting in seven studies being included: three quantitative and five qualitative studies (one study contained both eligible quantitative and qualitative data). Two of the quantitative studies could not be used in the data synthesis as they were judged to have a critical risk of bias and, in accordance with the protocol, were excluded from the meta‐analysis on the basis that they would be more likely to mislead than inform. The third quantitative study did not provide enough information enabling us to calculate an effect size and standard error. Meta‐analysis was therefore not possible. Following quality appraisal of the qualitative studies, three qualitative studies were judged to be of sufficient methodological quality. It was not possible to perform a qualitative thematic synthesis since in two of these studies, findings particular to special education class size were scarce. Therefore, only descriptive data extraction could be performed.

**Main Results:**

Despite the comprehensive searches, the present review only included seven studies published between 1926 and 2020. Two studies were purely quantitative (Forness, 1985; Metzner, 1926) and from the U.S. Four studies used qualitative methodology (Gottlieb, 1997; Huang, 2020; Keith, 1993; Prunty, 2012) and were from the US (2), China (1), and Ireland (1). One study, MAGI Educational Services (1995), contained both eligible quantitative and qualitative data and was from the U.S.

**Authors' Conclusions:**

The major finding of the present review was that there were virtually no contemporary quantitative studies exploring the effects of small class sizes in special education, thus making it impossible to perform a meta‐analysis. More research is therefore thoroughly needed. Findings from the summary of included qualitative studies reflected that to the special education students and staff members participating in these studies, smaller class sizes were the preferred option because they allowed for more individualised instruction time and increased teacher attention to students' diverse needs. It should be noted that these studies were few in number and took place in very diverse contexts and across a large time span. There is a need for more qualitative research into the views and experiences of teachers, parents, and school administrators with special education class sizes in different local contexts and across various provision models. But most importantly, future research should strive to represent the voices of children and young people with special needs since they are the experts when it comes to matters concerning their own lives.

## 1 PLAIN LANGUAGE SUMMARY

### 1.1 Little evidence exists on the effects of small class sizes in special education

Despite carrying out extensive literature searches, the authors of this review found only seven studies exploring the question of class size in special education. The authors therefore call for more research from quantitative and qualitative researchers alike, such that practitioners and administrators may find guidance in their endeavours to create the best possible school provisions for all children with special educational needs.

### 1.2 What is this review about?

While research on the relationship between general education class size and student achievement is plentiful, research on class size in special education is scarce, even though class size issues must be considered particularly important to students with special educational needs. This systematic review sought to examine the effects of small class sizes in special education on the academic achievement, socioemotional development and well‐being of children with special educational needs.

Furthermore, the review aimed to perform a qualitative exploration of the views of children, teachers and parents concerning class size conditions in special education.

A secondary objective was to explore how potential moderators (e.g. performance at baseline, age, and type of special educational need) affected the outcomes.

**What is the aim of this review?**

The objective of this Campbell systematic review was to synthesise data from existing studies to assess the impact of small class sizes in special education on students' academic achievement, socioemotional outcomes and well‐being.

### 1.3 What studies are included?

This review included seven studies, of which two were quantitative, four were qualitative, and one was both quantitative and qualitative. It was not possible to perform a meta‐analysis, nor a qualitative thematic synthesis. The included studies were critically assessed, coded for descriptive data, and narratively summarised.

One quantitative study was assessed to be of sufficient methodological quality following risk of bias assessment. Unfortunately, it was not possible to extract an effect size from this study since it did not report the required information and the study authors could not be contacted.

Three qualitative studies were assessed to be of sufficient methodological quality following qualitative critical appraisal.

### 1.4 What are the main findings of this review?

There are surprisingly few studies exploring the effects of small class sizes in special education on any outcomes. The included qualitative studies find that smaller class sizes are the most preferred option among students with special educational needs, their teachers and school principals. This is because of the possibilities afforded in terms of individualised instruction time and increased teacher attention to the needs of each student.

### 1.5 What do the findings of this review mean?

The impact of small class sizes in special education is under‐researched both within the quantitative and the qualitative literature.

Future research should aim to fill this knowledge gap from diverse methodological perspectives, paying close attention to the views of parents, teachers, administrators and, most importantly, the children and young people whose everyday lives are spent in the various special education provisions.

### 1.6 How up‐to‐date is this review?

Searches in bibliographic databases and EBSCO OPEN Dissertations were performed in April 2021, while the remaining searches for grey literature, hand searches in key journals, and citation tracking took place between January and May 2022.

## 2 BACKGROUND

### 2.1 Description of the condition

Class size reductions in general education are some of the most researched educational interventions in social science, yet researchers have not reached any final conclusions regarding their effects. While some researchers point to small and insignificant differences between varying class sizes, others find positive and significant effects of small class sizes on, for example, children's academic outcomes. In a previous Campbell Systematic Review on small class sizes in general education, Filges (2018) found evidence suggesting, at best, a small effect on reading achievement, whereas there was a negative, but statistically insignificant, effect on mathematics.

While research on the relationship between general education class size and student achievement is plentiful, research on class size in special education is scarce (see e.g., McCrea, 1996; Russ, 2001; Zarghami, 2004), even though class size issues must be considered particularly important to students with special educational needs. These students compose a highly diverse group, but they share a common need for special educational accommodations, which often entails additional instructional support in smaller units than what is usually provided in general education. Special education class sizes may vary greatly, both across countries and regions, as well as across different student groups, but will usually be small relative to general education classrooms. In most cases, placement in special education, as opposed to, for example, inclusion in general education, is based exactly on the child's need for close adult support in a smaller unit, where instruction can be tailored to the needs of each child and a calmer, more structured environment can be created. Following this, one may assume that there are advantages to small class sizes in special education, in that children are placed in a suitable environment with the support they need to thrive and learn (for a discussion of perceptions on the benefits of special education, see e.g., Kavale, 2000). However, there may also be challenges to small class sizes, for example, in terms of the opportunities available for building friendships.

It should be noted that class size in special education is connected to other structural factors such as, for example, student–teacher ratio and type of special education provision. In this review, we focus on class size since our main interest lies in exploring the specific mechanisms behind being in a smaller group. However, we have paid close attention to the relatedness and potential overlap between class size and concepts such as student/teacher ratio or caseload (for more about these concepts, see Description of the intervention). When it comes to the type of special education provision, we have included all types of settings where children with special educational needs are grouped together for instruction (i.e., segregated schools/classes/groups/units to which only students with special educational needs attend).

Finally, class size issues, both in general and in special education, are associated with ongoing discussions on educational spending and budgetary constraints. Hence, in school systems imposed with financial constraints, small class sizes in special education settings may be deemed too expensive. As a result, children with special educational needs may be placed in larger units with potential adverse effects on their learning and well‐being. At this point, there is however a lack of clarity as to the effects of small class sizes in special education on student academic achievement, socioemotional development, and well‐being. Inevitably, such lack of clarity is an obstacle for special educators and policymakers trying to make informed decisions. This highlights the policy relevance of the current systematic review, in which we examined the effects of small class sizes in special education on the academic achievement, socioemotional development, and well‐being of children with special educational needs. In working towards this aim, we planned to apply an approach consisting of both a statistical meta‐analysis (if possible from the studies found through our searches) and an exploration of the experiences of children, teachers, and parents with class size issues in special education, as reported in qualitative studies. We chose to include studies applying a qualitative methodology because the combination of quantitative and qualitative methods had the potential to provide a deeper insight into the complexity of class size questions in special education, including the voices of children and teachers who spend their everyday lives in special education contexts.

### 2.2 Description of the intervention

*Special education* in this review refers to educational settings designed to provide instruction exclusively for children with special educational needs. In such settings, both the instructional and physical classroom environment may be adjusted to accommodate the specific needs of the student group, as in the use of individual work tables and visual aids (pictograms) for children on the autism spectrum. We have included studies of all kinds of special education settings that are attended only by children with special educational needs (i.e., segregated special education settings as opposed to inclusion settings where children with and without special educational needs are taught together). We have included both part‐ and full‐time special education provisions (with an example of a part‐time provision being resource rooms attended by students with specific learning difficulties within one or more academic subjects). Furthermore, no limits have been imposed concerning the placement of special education provisions, that is, we have included both separate special schools and special education classes, units or resource rooms lodged within mainstream schools. We acknowledge that significant variations exist in special education provisions across time (e.g., due to new developments in pedagogical approaches and learning aids) and between (as well as within) countries, just as we are aware of the diversity between special education provisions, for example, in terms of how they are staffed and to which degree they are specialised to work with particular student groups. Our approach has therefore been to be inclusive in our search and screening process by not imposing limits on publication date or study location and by defining special education as all kinds of provisions where children with any type of special educational need are grouped together for instruction for any given amount of time (for our definition of what constitutes a special educational need, see Types of participants).

In this review, it is important to distinguish between the following terms: *class size*, *student–teacher ratio*, and *caseload*. *Class size* refers to the number of students present in a classroom at a given point in time. *Student–teacher ratio* refers to the number of students per teacher within a classroom or an educational setting. Furthermore, some studies may apply the term *caseload* which is typically defined as the number of students with individual education plans (IEPs) for whom a teacher serves as ‘case manager’ (Minnesota Department, 2000). In this review, the intervention is a small class size. Thus, studies only considering student–teacher ratios or caseloads are not eligible.

Our rationale for focusing on class size is based in the belief that although class size and student–teacher ratios or caseloads in special education are related, they involve somewhat different assumptions about how a small class size as opposed to a larger one might change the opportunities for students and teachers. With class size, the mechanism in play is based on assumptions about the dynamics of a smaller group and the belief that with smaller groups, teachers are better able to develop an in‐depth understanding of student needs through more focused interactions, better assessment, and fewer disciplinary problems (Ehrenberg, 2001; Filges, 2018). The size of the group in itself will often be of specific importance to students with special educational needs, for example, students diagnosed with sensory processing disorders, making them sensitive to noise and movement, or students with ASD who struggle with reading social cues in larger groups. For such students, being in a larger class would likely feel overwhelming and stressful, no matter the student–teacher ratio.

Student–teacher ratio and caseload are also of great importance, but do not take in the specific mechanisms of being in a smaller group which we find to be central in special education. We acknowledge the relatedness of these concepts to class size and are aware that terms may in some cases overlap. We paid attention to this when searching for studies by adding a search term for student–teacher ratio and when screening the studies.

It is possible that the intensity of the intervention, that is, the size of a change in class size and the initial class size from which this change is made, can play a role in determining the intervention effect. For intensity, the question is: how small does a class have to be to optimise the advantage? In general education for example, large gains are attainable when class size is below 20 students (Biddle, 2002; Finn, 2002), but gains are also attainable if class size is not below 20 students (Angrist, 1999; Borland, 2005; Fredriksson, 2013; Schanzenbach, 2007). It has been argued that the impact of class size reductions of different sizes and from different baseline class sizes is reasonably stable and more or less linear when measured per student (Angrist, 2009; Schanzenbach, 2007). Other researchers argue that the effect of class size is not only non‐linear but also non‐monotonic, implying that an optimal class size exists (Borland, 2005). Thus, the question of whether the size of a change in class size and the initial class size from which the change is made matters for the magnitude of intervention effects is still an open question. For this reason, we planned to include intensity (size of change in special education class size and initial class size) as a moderator if it was possible given the information presented in the included studies.

### 2.3 How the intervention might work

Due to the specialised and varied nature of special needs provision, issues of class size in this area are likely to be complex (Ahearn, 1995). However, small class sizes may promote student engagement and instructional individualisation, which is of particular importance to students with special educational needs. A research report from 1997 evaluating increases in resource room instructional group size in New York City public schools may serve to illustrate the importance of individualisation in special education (Gottlieb, 1997). The report indicated that increases in instructional group sizes from 5 to at most 8 students per teacher led to decreases in the reading achievement scores of resource room students. Resource room teachers reported diminished opportunities for sufficiently helping students. Furthermore, observations revealed little time spent on individual instruction.

Small class sizes may be better suited to address the potential physical and psychological challenges of students with special educational needs, for example, by providing closer adult‐child interaction, better accommodation of individual needs, and a more focused social interaction with fewer peers. Thus, smaller class sizes in special education may have a positive impact on both academic achievement and socioemotional development as well as on student well‐being at school.

On the other hand, small class sizes may limit the possibilities for finding compatible peers with whom to build friendships, hence leading to adverse effects on student's social and personal well‐being at school. This may also impact on the options available for building social skills, which are vital to, for example, students with autism‐spectrum‐disorders. Furthermore, small class sizes may lead to decreased variation in academic and social skills within the class, limiting the potential for positive peer effects on student academic learning and socioemotional development (e.g., learning from peers with more advanced academic skills).

As reflected in the above discussion about the potential benefits (or lack thereof) pertaining to smaller class sizes in special education, the effects of any given change in class size may occur both within the realm of academic achievement as well as across socioemotional domains (covering children's psychological, emotional, and social adjustment, as well as mental health) and in terms of student well‐being (defined as children's subjective quality of life, pleasant emotions, happiness, and low levels of stress and negative moods); each of these domains (academic achievement, socioemotional development, and well‐being) are therefore included as key outcomes in the present review.

### 2.4 Why it is important to do this review

As previously noted, there is a lack of clarity as to the impact of small class sizes in special education on student academic achievement, socioemotional development, and well‐being, making it difficult for special educators and policymakers to make informed decisions. Furthermore, class size alterations are associated with ongoing discussions on educational spending and budgetary constraints, highlighting the policy relevance of strengthening the knowledge base through a systematic review of the available literature.

Few authors have tried to review the available literature on special education class sizes, and these reviews have not followed rigorous, systematic frameworks, such as that applied in a Campbell systematic review. McCrea (1996) conducted a review on special education and class size including a sample of American studies. These studies pointed to some effects of class size on the learning environment in class as well as on student achievement and behaviour, especially at the elementary level. Furthermore, in an article exploring the class size literature, Zarghami (2004) examined the effects of appropriate class size and caseload on special education student academic achievement. The authors were not able to identify a single best way to determine appropriate class and group sizes for special education instruction. However, they pointed to the existence of well‐qualified teachers as an important factor in increasing student achievement. Finally, Ahearn (1995) analysed state special education regulations on class size/caseload in the U.S. and reviewed research on class size in general education and special education. The report showed that state requirements for class size/caseload in special education programmes were much more specific and complicated than those for general education, and that the specialised nature and variety of the services delivered to students with special educational needs, combined with the restrictions attributable to specific student disabilities, contributed to those complications. In line with the article by Zarghami (2004), Ahearn (1995) concluded that there was no single best way to determine class sizes for special education programmes, adding that the information available was inadequate.

The above mentioned reviews did not apply the extensive, systematic literature searches and critical appraisals that are performed in a Campbell systematic review. Furthermore, they date back 15 years or more, which means that they do not include newer developments in special education research. Therefore, we find that the present review fills a research gap by providing an up‐to‐date overview of what (little) research is available exploring the effects of small class sizes in special education and the views of children, parents, and teachers who experience different issues related to special education class size. In this sense, the main contribution of the review lies in shedding light on the fact that more research is still needed to gain knowledge into the complexities of class size in special education.

## 3 OBJECTIVES

The objective of this systematic review was to uncover and synthesise data from studies to assess the impact of small class sizes on the academic achievement, socioemotional development, and well‐being of students in special education. We also aimed to investigate the extent to which the effects differed among subgroups of students. Furthermore, we aimed to perform a qualitative exploration of the experiences of children, teachers, and parents with class size issues in special education.

## 4 METHODS

### 4.1 Criteria for considering studies for this review

#### 4.1.1 Types of studies

The screening of potentially eligible studies for this review was performed according to inclusion criteria related to types of study designs, types of participants, types of interventions, and types of outcome measures, all of which are described in the following sections (for the screening guide, see Supporting Information: Appendix 2). These criteria were also specified in the published protocol (Bondebjerg, 2021).

To summarise what is known about the possible causal effects of small special education class sizes, we included all quantitative study designs that used a well‐defined control or comparison group, that is, studies that compared outcomes for groups of students in smaller versus larger special education classes. This is further outlined in the section *Assessment of risk of bias in included studies*, and the methodological appropriateness of the included quantitative studies was assessed according to the risk of bias.

The quantitative study designs included in the review were:

1. Randomised and quasi‐randomised controlled trials (allocated at either the individual or cluster level, for example, class/school/geographical area etc.),
2. Non‐randomised studies (where allocation had occurred in the course of usual decisions, was not controlled by the researcher, and included a comparison of two or more groups of participants, that is, at least a treated group and a control group).

For non‐randomised studies, where the change in class size occurred in the course of usual decisions (e.g., due to policies mandating class size alterations), we assessed whether the authors demonstrated sufficient pre‐reatment group equivalence on key participant characteristics.

Studies using single group pre‐post comparisons were not included. Non‐randomised studies using an instrumental variable approach were also not included—see Supporting Information: Appendix 1 (*Justification of exclusion of studies using an instrumental variable (IV) approach*) for our rationale for excluding studies of these designs. A further requirement to all types of studies (randomised as well as non‐randomised) was that they were able to identify an intervention effect. Studies where, for example, small classes were present in one school only and the comparison group was larger classes at another school (or more schools for that matter), would not be able to separate the treatment effect from the school effect.

The treatment in this review was a small class size. To investigate the effects of small class sizes, we included studies that compared students in smaller classes with students in larger classes. This meant that we included both studies where the intervention consisted of a reduction in class size and studies where there was an increase in class size, since both types of studies (if robustly conducted) would allow us to compare the outcomes of children in smaller classes with those of children in larger classes. We only included studies that used measures of class size and measures of outcome data at the individual or class level. We excluded studies that relied on measures of class size and measures of outcomes aggregated to a level higher than the class (e.g., school or school district).

In addition to exploring the causal effects of small class sizes in special education through an analysis of quantitative studies meeting the criteria above, we aimed to gain qualitative insight into the experiences of children, teachers, and parents with class size issues in special education contexts. To this end, we included all types of empirical qualitative studies that collected primary data and provided descriptions of main methodological issues such as informant selection, data collection procedures, and type of data analysis. Eligible qualitative studies may apply a wealth of data collection methods, including (but not limited) to participant observations, in‐depth interviews, or focus groups.

If we found mixed‐methods studies combining qualitative and quantitative data collection procedures, we assessed whether the quantitative data were eligible for inclusion in the quantitative part of the review (i.e., the quantitative data met the criteria imposed on studies exploring causal relationships), and whether the qualitative data met the criteria imposed on qualitative studies. If a study contained both eligible quantitative and qualitative data, it was included for both quantitative and qualitative quality assessment and data extraction and was counted in both categories. If there were only eligible quantitative data, the study was included only in the quantitative part of the review, and vice‐versa for qualitative studies. That is, mixed methods studies were not treated as a separate category, but were included if either their quantitative or their qualitative research components met the inclusion criteria for quantitative or qualitative studies, respectively.

#### 4.1.2 Types of participants

The review included studies of children with special educational needs in grades K‐12 (or the equivalent in European countries) in special education. Studies that met the inclusion criteria were accepted from all countries. In this review, we excluded children in home‐ or preschool as well as children placed in treatment facilities.

Some controversy exists regarding the definition of what constitutes a special educational need (Vehmas, 2010; Wilson, 2002). In this review, we were guided by the definition from the US Individuals with Disabilities Education Act (IDEA), in which special needs are divided into 13 different disability categories^1^:

- specific learning disability (covers challenges related to a child's ability to read, write, listen, speak or do math, e.g., dyslexia or dyscalculia),
- other health impairment (covers conditions limiting a child's strength, energy, or alertness, e.g., ADHD),
- autism spectrum disorder (ASD),
- emotional disturbance (may include e.g., anxiety, obsessive‐compulsive disorder and depression),
- speech or language impairment (covers difficulties with speech or language, e.g., language problems affecting a child's ability to understand words or express herself),
- visual impairment (covers eyesight problems, including partial sight and blindness),
- deafness (covers instances where a child cannot hear most or all sounds, even with a hearing aid),
- hearing impairment (refers to a hearing loss not covered by the definition of deafness),
- deaf‐blindness (covers children suffering from both severe hearing and vision loss),
- orthopaedic impairment (covers instances when a child has problems with bodily function or ability, as in the case of cerebral palsy),
- intellectual disability (covers below‐average intellectual ability),
- traumatic brain injury (covers brain injuries caused by accidents or other kinds of physical force),
- multiple disabilities (children with more than one condition covered by the IDEA criteria).

While the above listed criteria provided useful guidance, we were fully aware that they should not be conceived as exhaustive, nor as clear‐cut definitions of what constitutes special educational needs. Therefore, we did not restrict ourselves to only include studies that defined their participants with these terms or which provided detailed information about types of special educational needs. Rather, we included all studies where the participating students received instruction in segregated special education settings (since we took placement in such settings to necessarily indicate a need for specialised educational support) and planned to explore the potential variation between different groups of students, if possible from the included studies.

#### 4.1.3 Types of interventions

In this review, we were interested in investigating whether small class sizes in special education resulted in better academic achievement, socioemotional development, and well‐being for students in special education when compared to larger class sizes. To answer this question, we included studies where special education class size was altered either as a result of a deliberate experiment (where class size was directly manipulated by researchers) or as a result of a naturally occurring change in class size arising due to, for example, the implementation of a new class size policy. This meant that the intervention of interest to this review was a change in special education class size allowing for a comparison between students in smaller classes versus students in larger classes. That is, the question of the effect of small class sizes could be investigated both by looking at studies where class size was reduced and where class size was increased, provided that the studies used a control or comparison group of students in smaller or larger special education classes than the treated group.

The more precisely a class size is measured, the more reliable the findings of a study will be. Studies only considering the average class size measured as student–teacher ratio within a school (or at higher levels) were not eligible. Studies where the intervention was the assignment of an extra teacher (or teaching assistants or other adults) to a class were not eligible. The assignment of additional teachers (or teaching assistants or other adults) to a classroom is not the same as changing the size of the class, and this review focused exclusively on class size. We acknowledged that class size can change per subject or eventually vary during the day, which is why the precision of the class size measure was recorded if possible.

*Special education* refers to settings where children with special educational needs are taught in classes segregated from general education students. These classes may be composed of children with similar special educational needs (such as classes specifically for children with ASD) or they may consist of mixed groups of children with diverse special educational needs. In such settings, the instructional environment is adjusted to accommodate the specific needs of the student group. In the present review, *special education* was thus defined as any given group composition consisting of only children with special educational needs. In some studies, *special education* was also referred to as, for example, *segregated placement* or *resource room*. Special education could be full‐time or part‐time (e.g., in the form of resource rooms attended by students for parts of the day). We included studies of all kinds of special education.

#### 4.1.4 Types of outcome measures

For quantitative studies, only valid and reliable outcomes that had been used on different populations were eligible.

##### Primary outcomes

Academic achievement (measured with e.g., the Woodcock‐Johnson III Tests of Achievement, Mather, 2001), socioemotional development and adjustment (measured with e.g., The Strengths and Difficulties Questionnaire [SDQ], Goodman, 2001), and well‐being (measured with e.g. The Perceived Competence Scale for Children, Harter, 1982) were categorised as primary outcomes.

##### Secondary outcomes

In addition to the primary outcomes, we considered school completion rates as a secondary outcome. Furthermore, we included validated measures of student classroom behaviour, such as structured observations of student engagement, on‐task behaviour, and disruptive behaviour (measured with e.g., The Code for Instructional Structure and Student Academic Response [CISSAR], Greenwood, 1978).

Studies were only included if they considered at least one of the primary or secondary outcomes.

##### Duration of follow‐up

The review aimed to include follow‐up measures at any given point if meaningful based on the objectives for the review. However, none of the included studies reported outcomes past the end of the intervention.

##### Qualitative outcomes

For the qualitative analysis, we were interested in exploring the experiences of children, teachers, and parents with special education class sizes, as they presented themselves through, for example, in‐depth qualitative interviews or participant observations. Relevant data could stem from, for example, interviews with teachers on their perceptions of childrens' academic achievement and well‐being in small versus large special education classes, or their experiences with ensuring student engagement and attention under different class sizes. We did not define a list of outcomes in advance, but remained open to what presented itself as important to children, teachers, and parents concerning special education class sizes.

##### Types of settings

In this review, we included studies of children with special educational needs placed in any special education setting. We excluded studies of children in home‐ or preschool as well as children placed in treatment facilities.

### 4.2 Search methods for identification of studies

Relevant quantitative and qualitative studies were identified through searches in electronic databases, grey literature resources, and Internet search engines, as well as through hand‐searches in specific targeted journals and citation‐tracking. We searched for both published and unpublished literature and screened references in English, Danish, Swedish, and Norwegian.

Locating qualitative research presents the reviewer with particular challenges since existing search strategies have largely been developed for and applied to the quantitative literature (Frandsen, 2016). As of yet, not all databases have implemented rich qualitative vocabularies or specific structures tailored to accommodate qualitative literature searches. Furthermore, screening on title and abstract may prove challenging since titles and abstracts in qualitative studies are sometimes more focused on content than on issues of methodology (Ibid). Attempts have been made to develop tools specifically designed for qualitative literature searches as an answer to the perceived difficulties in using such existing tools as the PICO(s) framework (**P**opulation, **I**ntervention, **C**omparison (or control), **O**utcome, and **S**tudy design and type). Cooke (2012), for example, present the SPIDER search strategy which attempts to adapt the PICO components to make them more suitable for qualitative research. The SPIDER strategy contains the following components: **S**ample, **P**henomenon of **I**nterest, **D**esign, **E**valuation, and **R**esearch type. In the study by Cooke (2012), two systematic searches are performed, using first the PICO framework and then the SPIDER tool. The results show that the PICO search strategy generates a large number of hits, while the SPIDER tool leads to fewer hits, with the potential advantage of greater specificity. This means that the SPIDER tool may be more precise and easier to manage in terms of the amount of references for screening, however carrying the risk of missing studies.

In this review, we applied elements of the PICO(s) framework to search for both quantitative and qualitative studies by adding both quantitative and qualitative methodological terms in the search string, as well as by carefully looking for both types of studies in our grey literature and hand‐searches. By choosing this strategy, we prioritised the breadth and comprehensiveness of our search (sensitivity) which seemed the most appropriate choice given the anticipated low number of studies exploring class size effects particular to special education. Given the low number of studies found in the searches, we are convinced that our comprehensive approach was the best choice for this particular review topic.

#### 4.2.1 Electronic searches

The following bibliographic databases were searched in April 2021:

- ERIC (EBSCO‐host, 1966–2021)
- Academic Search Premier (EBSCO‐host, 1931–2021)
- EconLit (EBSCO‐host, 1969–2021)
- APA PsycINFO (EBSCO‐host, 1890–2021)
- SocINDEX (EBSCO‐host, 1895–2021)
- International Bibliography of the Social Sciences (ProQuest, 1951–2021)
- Sociological Abstracts (ProQuest, 1952–2021)
- Web of Science (Clarivate, Science Citation Index Expanded, 1900–2021, and Social Sciences Citation Index, 1956–2021)

##### Description of search string

The search string was based on the PICO(s)‐model, and contained three concepts of which we developed three corresponding search facets: population, intervention, and study type/methodology. The search string includes searches in title, abstract, and subject terms for each facet. To increase the sensitivity of the search, we also searched in full text for the intervention terms. The subject terms in the facets were selected according to the thesaurus or subject term index on each database.

##### Example of a search string

The search string below from the ERIC database exemplifies the search which followed this structure:

- Search 1–4 covered the population,
- Search 5–9 covered the intervention,
- Search 10–16 covered the study type/methodology terms,
- Search 17 combined the three aspects.

**Table:** 

**Search**	**Terms**
S17	S4 AND S9 AND S16
S16	S10 OR S11 OR S12 OR S13 OR S14 OR S15
S15	DE (‘Qualitative Research’ OR ‘Ethnography’ OR ‘Case Studies’ OR ‘Evaluation Methods’ OR ‘Field Studies’ OR ‘Focus Groups’ OR ‘Interviews’ OR ‘Mixed Methods Research’ OR ‘Naturalistic Observation’ OR ‘Participant Observation’ OR ‘Classroom Observation Techniques’ OR ‘Observation’ OR ‘Action Research’)
S14	AB (qualitative* OR ethnograp* OR ‘case stud*’ OR evaluation* OR ‘focus group*’ OR interview* OR ‘mixed method*’ OR observation*)
S13	TI (qualitative* OR ethnograp* OR ‘case stud*’ OR evaluation* OR ‘focus group*’ OR interview* OR ‘mixed method*’ OR observation*)
S12	DE (‘Effect Size’ OR ‘Control Groups’ OR ‘Experimental Groups’ OR ‘Experiments’ OR ‘Matched Groups’ OR ‘Quasiexperimental Design’ OR ‘Randomized Controlled Trials’ OR ‘Comparative Testing’ OR ‘Intervention’)
S11	AB (effect* OR trial* OR experiment* OR ‘control group*’ OR random* OR impact* OR compar* OR difference*)
S10	TI (effect* OR trial* OR experiment* OR ‘control group*’ OR random* OR impact* OR compar* OR difference*)
S9	S5 OR S6 OR S7 OR S8
S8	DE (‘Class Size’ OR ‘Small Classes’ OR ‘Teacher Student Ratio’)
S7	TX (group* OR class*) N5 (size*)
S6	AB (group* OR class*) AND AB (size* OR ratio*)
S5	TI (group* OR class*) AND TI (size* OR ratio*)
S4	S1 OR S2 OR S3
S3	DE (‘Special Needs Students’ OR ‘Special Schools’ OR ‘Residential Schools’ OR ‘Educationally Disadvantaged’ OR ‘Developmental Delays’ OR ‘Students with Disabilities’ OR ‘Special Classes’ OR ‘Special Education’ OR ‘Self Contained Classrooms’ OR ‘Resource Room’)
S2	AB (special*) AND AB (need* OR education OR child* OR student* OR pupil*)
S1	TI (special*) AND TI (need* OR education OR child* OR student* OR pupil*)

##### Limitations of the search string

We did not restrict our searches based on publication date or language. In screening and processing the references found, we were however limited by the language proficiencies available on the review team which allowed us to consider studies published in English, Danish, Norwegian, and Swedish.

#### 4.2.2 Searching other resources

##### Hand‐search

We implemented hand‐searches in key journals to identify references that were poorly indexed in the bibliographic databases and to ensure coverage of references that were published, but had not yet been indexed. We hand‐searched individual tables of content of respective issues of the chosen journals going back to 01/01/2015.

Our selection of journals to hand‐search was based on the frequency of journals identified in our pilot searches during the design phase of the search string. The following journals were selected:

- *Behavioral Disorders*
- *Journal of Autism & Developmental Disorders*
- *Exceptional Children*
- *Learning Disability Quarterly*
- *International Journal of Disability, Development & Education*
- *Remedial and Special Education*
- *Journal of Speech, Language, and Hearing Research*
- *British Journal of Special Education*
- *Learning Disabilities Research & Practice*
- *Journal of Intellectual Disability Research*
- *European Educational Research Journal*

##### Searches for unpublished literature

Most of the resources searched for unpublished literature contained multiple types of unpublished literature. For the sake of transparency, we have divided the resources into categories based on the most prevalent type of literature in the resource.

*Searches for dissertations and theses in English:*

- EBSCO Open Dissertations (EBSCO‐host)

*Searches for working papers and conference proceedings in English:*

- Google Scholar—https://scholar.google.com/
- Social Science Research Network—https://www.ssrn.com/index.cfm/en/
- OECD iLibrary—https://www.oecd-ilibrary.org/
- NBER working paper series—http://www.nber.org
- American Educational Research Association (AERA)—https://www.aera.net/

*Search for Reports and on‐going studies in English:*

- Google searches—https://www.google.com/
- Best Evidence Encyclopaedia—http://www.bestevidence.org/
- Social Care Online—https://www.scie-socialcareonline.org.uk/

*Searches for dissertations, theses, working papers and conference proceedings in Danish, Swedish, and Norwegian:*

- Forskning.ku—Academic publications from the University of Copenhagen—https://forskning.ku.dk/soeg/
- AAU Publications—Academic publications from the University of Aarhus https://pure.au.dk/portal/da/organisations/8000/publications.html
- SwePub ‐ Academic publications at Swedish universities—http://swepub.kb.se/se/
- NORA ‐ Norwegian Open Research Archives—http://nora.openaccess.no/
- DIVA—Swedish Digital Scientific Archives—http://www.diva-portal.org/smash/
- Skolporten—Swedish Dissertations—https://www.skolporten.se/forskning/

*Searches for reports and on‐going studies in Danish, Swedish, and Norwegian:*

- CORE—research outputs from international repositories ‐ https://core.ac.uk/
- Google searches—https://www.google.com/

##### Search for systematic reviews

We searched for systematic reviews through the following resources:

- Campbell Journal of Systematic Reviews—https://campbellcollaboration.org/
- Cochrane Library—https://www.cochranelibrary.com/
- Centre for Reviews and Dissemination Databases—https://www.crd.york.ac.uk/CRDWeb/
- EPPI‐Centre Database of Education Research—https://eppi.ioe.ac.uk/webdatabases/Intro.aspx?ID=6

##### Citation‐tracking and snowballing methods of systematic reviews

We performed citation‐tracking on systematic reviews identified in the protocol stage and through the search process to identify additional relevant references. The following reviews/research overviews were processed using both forward and backward citation‐tracking: Ahearn, 1995; McCrea, 1996; Zarghami, 2004.

##### Citation‐tracking and snowballing methods of individual references

We had planned to select the most recently published and the most cited key references for citation‐tracking, with the expectation that we would select approximately 20 references (10 recent, 10 most cited). This approach was made impossible by the low number of relevant references found during the search process. We therefore chose to perform citation‐tracking on the included references: Forness, 1985; Gottlieb, 1997; Huang, 2020; Keith, 1993a; Metzner, 1926; Prunty, 2012. It was not possible to perform citation‐tracking on the study from MAGI Educational Services, Inc., 1995 since it did not contain a reference list.

##### Contact to experts

We had planned to contact study authors if we found references to or mentions of ongoing studies in screened publications, but this did not occur during the search and screening process. Furthermore, the searches did not locate any particular individual experts or institutions that we could reach out to for more information on published or unpublished studies covering the subject matter.

A complete overview of the search strings used and the resulting references found for each electronic database, as well as search terms and hits for the grey literature resources, and results from the hand‐searches can be found in the appendix. Database searches were performed in April 2021. Searches for grey literature, hand‐search in key journals, and citation‐tracking took place between January and May 2022 (with the exception of the search in EBSCO OPEN Dissertations which was performed in April 2021, simultaneous with the database searches).

### 4.3 Data collection and analysis

#### 4.3.1 Selection of studies

Under the supervision of review authors, two review team assistants first independently screened titles and abstracts to exclude studies that were clearly irrelevant. Studies considered eligible by at least one assistant or studies where there was insufficient information in the title and abstract to judge eligibility were retrieved in full text. The full texts were subsequently screened independently by two review team assistants under the supervision of the review authors. Any disagreement of eligibility was resolved by the review authors. Screening on both title/abstract and full text was performed using EPPI‐Reviewer 4 software (Thomas, 2022). Exclusion of studies that otherwise might be expected to be eligible was documented (see Excluded studies).

None of the review authors were blind to the authors, institutions, or journals responsible for the publication of the articles.

#### 4.3.2 Data extraction and management

Two review authors independently coded and extracted data from included studies. Coding sheets for quantitative and qualitative studies were piloted and revised as necessary. For the included quantitative studies, data was extracted regarding school setting and location, participant characteristics (for children: type of special need, age, ethnic/cultural/language background, SES, gender, and for teachers: education and experience), study design, class size information (including size and duration of class size alteration), type and format of data, outcome measurement, sample size, and effect size information (see Table 1 for the full data extraction sheet filled out with data from the included quantitative studies). From the included qualitative studies, we extracted information pertaining to the school setting and location, class size conditions, study design, theoretical perspective of the study, research objectives, student information (age, gender, SES, type of special need), and teacher and parent characteristics, if relevant (see Table 2 for the full data extraction sheet filled out with data from the included qualitative studies).

#### 4.3.3 Assessment of risk of bias in included studies

We did not locate any randomised studies. Therefore, included quantitative studies were assessed for risk of bias using the model ROBINS–I, developed by members of the Cochrane Bias Methods Group and the Cochrane Non‐Randomised Studies Methods Group (Sterne, 2016a). We used the latest template for completion (which was the version of 19 September 2016). The ROBINS‐I tool is based on the Cochrane RoB tool for randomised trials, which was launched in 2008 and modified in 2011 (Higgins, 2011a).

The ROBINS‐I tool covers seven domains (each with a set of signalling questions to be answered for a specific outcome) through which bias might be introduced into non‐randomised studies:

(1) bias due to confounding;
(2) bias in selection of participants;
(3) bias in classification of interventions;
(4) bias due to deviations from intended interventions;
(5) bias due to missing outcome data;
(6) bias in measurement of the outcome;
(7) bias in selection of the reported result.

The first two domains address issues before the start of the interventions and the third domain addresses classification of the interventions themselves. The last four domains address issues after the start of interventions and there is substantial overlap for these four domains between bias in randomised studies and bias in non‐randomised studies (although signalling questions are somewhat different in several places, see Sterne, 2016b and Higgins, 2019).

Non‐randomised study outcomes are rated on a ‘Low/Moderate/Serious/Critical/No Information’ scale on each domain. The level ‘Critical’ means that the study (outcome) is too problematic in this domain to provide any useful evidence on the effects of the intervention and is excluded from the data synthesis.

We discontinued the assessment of a non‐randomised study outcome as soon as one domain in the ROBINS‐I was judged as ‘Critical’. ‘Serious’ risk of bias in multiple domains in the ROBINS‐I assessment tool could also lead to a decision of an overall judgement of ‘Critical’ risk of bias for that outcome, leading the study to be excluded from the data synthesis.

##### Confounding

An important part of the risk of bias assessment of non‐randomised studies is consideration of how the studies deal with confounding factors. Systematic baseline differences between groups can compromise comparability between groups. Baseline differences can be observable (e.g., age and gender) and unobservable (to the researcher; e.g., childrens’ motivation and ‘ability’). There is no single non‐randomised study design that always solves the selection problem. Different designs represent different approaches to dealing with selection problems under different assumptions, and consequently require different types of data. There can be particularly great variation in how different designs deal with selection on unobservables. The ‘adequate’ method depends on the model generating participation, that is, assumptions about the nature of the process by which participants are selected into a programme.

As there is no universally correct way to construct counterfactuals for non‐randomised designs, we looked for evidence that identification was achieved, and whether the authors of the primary studies justified their choice of method in a convincing manner by discussing the assumptions leading to identification (the assumptions that made it possible to identify the counterfactual). Preferably, the authors should make an effort to justify their choice of method and convince the reader that the special needs students exposed to different class sizes were comparable.

In addition to unobservables, we identified the following observable confounding factors to be the most relevant for this review: performance at baseline, age of the child (chronological age and/or developmental age, if reported), category of special educational need and functional level, and socioeconomic background. In each study, we assessed whether these factors had been considered, and in addition we assessed other factors likely to be a source of confounding within the individual included studies.

##### Importance of pre‐specified confounding factors

The motivation for focusing on performance at baseline, age of the child, category of special educational need and functional level, and socioeconomic background, is outlined below.

Performance at baseline is a highly relevant confounding factor to consider, since students with special educational needs constitute a highly diverse population. There may be large achievement differences between children in special education classes, even when the children are of equal age and enroled in similar special education classes at the same grade level. This is true both when comparing children with different special educational needs profiles and children diagnosed with similar functional levels. This highlights the need for researchers to pay close attention to the risk of confounding due to achievement differences present at baseline.

The reason for including age as a pre‐specified confounder is that the needs of children change as they grow older. Young children are often more dependent on stimulating adult‐child interactions and have higher support needs, both academically and in terms of behavioural/emotional support. Therefore, to be sure that an effect estimate is a result from a comparison of groups with no systematic baseline differences, it is important to control for the students' age. In this review, it is important to both consider chronological age and developmental age, if this is reported.

As can be seen in the definition of special educational needs, the categories cover a very broad range of disabilities and functional levels. It is possible that special education students with some diagnoses or degrees of impairment require, for example, an increased need for individual support and close adult‐child interaction, or they may have an inability to cope in larger groups of children due to difficulties in sensory processing. Therefore, the special needs category and impairment level are important confounding variables.

Finally, a large body of research documents the impact of parental socioeconomic background on almost all aspects of childrens' development (e.g., Renninger, 2006), which is why we find it to be common place to include this as a potential confounding factor.

##### Effect of primary interest and important co‐interventions

We were mainly interested in the effect of actually participating in the intervention (in this case, receiving instruction in a smaller as opposed to a larger special education class), that is, the treatment on the treated effect (TOT). The risk of bias assessments were therefore carried out in relation to this specific effect. The risk of bias assessments considered adherence to intervention and differences in additional interventions (‘co‐interventions’) between intervention groups. Important co‐interventions we considered were other types of classroom support available to children with special educational needs, for example, software packages for children suffering from dyslexia. Furthermore, additional teachers or teacher aides in a classroom were considered an important co‐intervention.

##### Assessment

At least two review authors independently assessed the risk of bias for each relevant outcome from the included studies (see Table 3 for the risk of bias assessment of included quantitative studies).

#### 4.3.4 Measures of treatment effect

##### Continuous outcomes

For continuous outcomes, such as standardised reading tests, we planned to calculate effect sizes with 95% confidence intervals, where means and standard deviations were available. If means and standard deviations were not available, we intended to calculate standardised mean differences (SMD) from *F*‐ratios, *t*‐values, *χ* ^2^ values, and correlation coefficients, where available, using the methods suggested by Lipsey, 2001. Hedges' *g* would be used for estimating SMD. If insufficient information was reported in the studies, we had planned to request this information from the principal investigators. However, the only study where it was relevant to calculate an effect size lacked the information necessary for us to perform calculations; and since the study was from 1926, it was not feasible to contact the principal investigators for more information.

##### Dichotomous outcomes

For dichotomous outcomes, such as children passing or failing a test, we had planned to calculate odds ratios with 95% confidence intervals. However, none of the included studies contained dichotomous outcomes.

#### 4.3.5 Unit of analysis issues

We planned to take into account the unit of analysis of the studies to determine whether individuals were randomised in groups (i.e., cluster‐randomised trials), whether individuals may have undergone multiple interventions, whether there were multiple treatment groups, and whether several studies were based on the same data source.

##### Cluster‐randomised trials

There were no cluster‐randomised trials.

##### Multiple intervention groups and multiple interventions per individual

There were no studies with multiple intervention groups or multiple interventions per individual.

##### Multiple studies using the same sample of data

There were no studies using the same sample of data.

##### Multiple time points

There were no studies reporting on multiple time points.

#### 4.3.6 Dealing with missing data

Missing data and attrition rates in individual studies was assessed using the risk of bias tool. If summary data was missing, it was our plan to contact the study authors; this however turned out not to be feasible, since the only study where it was relevant to derive missing data was from 1926. Our options were therefore limited to reporting the study results in as much detail as possible based on the information available in the publication itself.

#### 4.3.7 Assessment of heterogeneity

We were unable to assess heterogeneity among primary outcome studies as no meta‐analysis could be performed.

#### 4.3.8 Assessment of reporting biases

Reporting bias refers to both publication bias and selective reporting of outcome data and results. Selective reporting was dealt with in the risk of bias assessment. Had we found sufficient studies, we would have used funnel plots for information about possible publication bias (Higgins, 2011b).

#### 4.3.9 Data synthesis

In the protocol for the review (Bondebjerg, 2021), we proposed a quantitative data synthesis based on standard procedures for conducting systematic reviews using meta‐analytic techniques. Studies that were coded ‘critical risk of bias’ were not included in the data synthesis. There were no studies to include in a meta‐analysis.

We aimed to use findings from qualitative studies to address and extend questions related to our effectiveness review, broadening the scope of the review to also include the lived experiences of children, teachers, and parents who spend their everyday lives in special education settings under different class size arrangements. As detailed in the review protocol (Bondebjerg, 2021), we planned to perform a thematic synthesis following the procedures presented in Thomas, 2008, but due to the limited number of studies, this was not a feasible approach. We therefore chose to present findings from each included study separately in the form of study abstracts.

#### 4.3.10 Subgroup analysis and investigation of heterogeneity

No studies were available for a meta‐analysis.

#### 4.3.11 Sensitivity analysis

No studies were available for a meta‐analysis.

##### Treatment of qualitative research

We included all types of empirical studies that collected qualitative data and provided descriptions of main methodological issues such as informant selection, data collection procedures, and type of data analysis. If an included quantitative study contained relevant qualitative data, these were treated in the same way as other qualitative studies and were considered for inclusion in the qualitative synthesis.

###### Critical appraisal of qualitative studies

All qualitative studies were appraised by two reviewers to assess whether or not they should be included in the thematic synthesis. Studies were double‐coded, after which the two reviewers discussed their assessments and reached a final conclusion on whether to include a given study in the synthesis. We only included studies for synthesis that paid sufficient attention to qualitative research standards for credibility, transferability, dependability, and confirmability (Hannes, 2011). We critically appraised qualitative studies using an adapted version of the JBI Critical Appraisal Checklist for Qualitative Research, developed by the Joanna Briggs Institute (Joanna Briggs Institute, 2017; Lockwood, 2015). This checklist includes 10 questions that lead to an overall appraisal of ‘Include’, ‘Exclude’, or ‘Seek further info’. The 10 questions take integral parts of the qualitative methodological process into consideration, such as the congruity between the choice of research methodology and the research objectives, the influence of the researcher on the research, and the flow of conclusions from the analysis or interpretation of data. In the original checklist, the questions are checked in boxes indicating ‘Yes’, ‘No’, ‘Unclear’ or ‘Not applicable’. In this review, reviewers were further required to justify their choice of ‘Yes’, ‘No’, ‘Unclear’ or ‘Not applicable’ in a comment box. This was done by importing the checklist into EPPI‐Reviewer 4 (Thomas, 2022) and adding comment boxes. Reviewers were also required to justify their overall appraisal assessment. The reason for demanding justifications in addition to ticking the boxes was founded on a wish to both ensure high methodological rigour and detail in the assessment. All critical appraisals of qualitative studies were performed in EPPI‐Reviewer 4 (Ibid.) and the full consensus ratings are shown in Table 4.

## 5 RESULTS

### 5.1 Description of studies

Despite the comprehensive searches, the present review only included seven studies published between 1926 and 2020. Two studies had eligible quantitative data (Metzner, 1926; Forness, 1985) and were from the U.S. Four studies used qualitative or mixed methods methodology and contained eligible qualitative data (Gottlieb, 1997; Huang, 2020; Keith, 1993a; Prunty, 2012); these studies were from the U.S. (2) China (1) and Ireland (1). One study, MAGI Educational Services, Inc., 1995 (from the U.S.) contained both eligible quantitative and qualitative data and was therefore included as both a quantitative and a qualitative study. Tables 1 and 2 provide an overview of the main characteristics for the seven included studies.

#### 5.1.1 Results of the search

**Figure 1:**
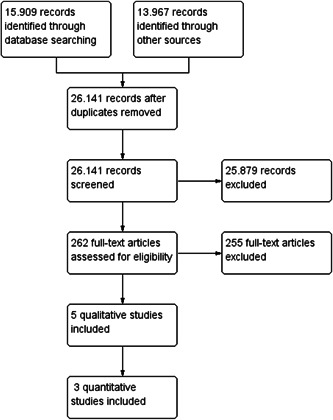
Please note that one study (MAGI Educational Services, year) was included as both a quantitative and qualitative study and is therefore counted in both categories.

Figure 1 shows a flow diagram for the search. Nine international bibliographic databases + EBSCO Dissertations were searched in April 2021. In addition, extensive searches for grey literature in international and Nordic resources, hand‐searches in 11 core journals, and citation‐tracking and snowballing were performed in the period from January to May 2022. All searches performed are documented in Supporting Information: Appendices 3–6.

After excluding duplicates, we found 26,141 potential records (bibliographic databases: 15,909, grey literature: 6955, hand searches: 6513, and citation‐tracking/snowballing: 499).

All 26,141 records were screened based on title and abstract, and 262 records were retrieved and screened in full text. Of these, 255 did not fulfill the screening criteria and were excluded.

Seven studies (reported in seven papers) met the inclusion criteria and were quality appraised and data‐extracted by the review authors. Descriptive details for the seven included studies are given in Tables 1 and 2.

#### 5.1.2 Included studies

The two studies containing only eligible quantitative data were published in 1926 and 1985, respectively. Metzner (1926), was an experimental study in which children with mental retardation received instruction in classes of varying sizes (treated were three classes with 15 students, three classes with 20 students, three classes with 25 students, and three classes with 30 students; controls were 12 classes with 22 students). Outcomes included the Pressy Reading Test and the Stanford Achievement Test for Grades 2 and 3. Forness (1985) explored the effects of class size on attention, communication, and disruptive behaviour of children with mild mental retardation. The children attended five small classes (10–13 students), 14 medium classes (14–16 students), and seven large classes (18–21 students). Outcomes consisted of observations of classroom behaviour in four categories: communication, attention, no attention, and disruption.

The four qualitative or mixed methods studies which contained only eligible qualitative data were published between 1993 and 2020 and had diverse research objectives, research designs, and types of data. Gottlieb (1997) was a mixed methods evaluation study using questionnaires, observations, interviews, and student achievement data. The research objective was to assess the impact of increases in instructional group sizes in resource rooms and speech services in the New York City Public Schools. Huang (2020) was a dissertation based on semi‐structured interviews with 32 special education teachers in China. The research objective for this study was to investigate Chinese special education teachers' perceptions and practices related to individualising or adapting instruction for students with intellectual and developmental disabilities, including their perceptions of potential barriers to such adaptation (of which large class sizes was one). Keith (1993a) was a mixed methods research report that investigated Virginia special education program standards, focusing on local applications of the standards for class size and class mix and the effect of varying class size and class mix on student outcomes. The report was based on interviews, observations, document reviews, and survey data. Prunty (2012) was a qualitative study based on focus group and individual interviews with 38 children and young people with special educational needs eliciting their views on mainstream and special education placement.

Finally, MAGI Educational Services, Inc. (1995) was an article containing both eligible quantitative and qualitative data from the New York Class Size Research Study. MAGI Educational Services, Inc. (1995) reported on two different studies: one was a descriptive mixed‐methods study based on data from 17 randomly selected upstate districts and 10 randomly selected New York City Community School Districts, with data collection consisting of document review, focus groups, public hearings, and surveys of key informants. The second study referred to as the observation study was a quasi‐experimental study in which students with special needs within segregated special education were observed in two class size conditions (12:1 and 15:1). Two standardised observational instruments were used: The Code for Instructional Structure and Student Academic Response (MS‐CISSAR), and The Instructional Environment System (TIES II).

**Table 1:** Data extraction: Quantitative studies.

Author	Title	Language	Outlet (journal name/other outlet/dissertation/unclear)	Year	Study location	Type of school and educational setting	Type of special need	Child age (mean and range)
Metzner (1926)	Size of class for mentally retarded children	English	The Training School Bulletin	1926	Detroit, U.S.	Type of school not specified, but setting is probably half‐time.	Mental retardation	Approximately an average of 10.8, range not reported.
Forness (1985)	Effects of class size on attention, communication, and disruption of mildly mentally retarded children	English	American Educational Research Journal	1985	California, U.S.	Not specified, but it is probable that all students spent more than half their day in special class, and that regular class integration was limited to non‐academic classroom periods during the afternoons.	Mildly mentally retarded (educable)	Mean age in small classes 12.3; medium classes 11.0; large classes 11.2; overall 11.3.
MAGI Educational Services, Inc. (1995)	Results of a Statewide Research Study on the Effects of Class Size in Special Education	English	Class Size Research Bulletin	1995	New York, U.S.	Modified Instructional Services (MIS) I classes which covered classes for students who required instructional services in a special class with opportunities for mainstreaming. Students could supposedly spend both full‐time or less in these classes, depending on pull‐out services or involvement with mainstream classrooms.	The majority of MIS I students were classified as learning disabled.	Not reported, but both elementary and secondary students.

Author	Child ethnic, cultural, and language background	Child SES	Child gender	Teacher education and experience	Study design	Class size	Intensity (size of reduction)	Duration of class size reduction
Metzner (1926)	Not specified	Not specified	Varies between 47.5% and 71.1% boys in treatment groups; in control group 64% boys.	Not specified		Treated are three classes with 15 students, three classes with 20 students, three classes with 25 students, and three classes with 30 students; controls are 12 classes with 22 students.	A reduction of 2 and 7 and an increase of 3 and 8.	180 days
Forness (1985)	Not specified	Not specified	Small classes: 50% male; medium classes 58% male; large classes 56% male; overall 56% male.	Not specified		5 small classes (10–13), 14 medium classes (14–16), and 7 large classes (18–21).	Mean difference between small and medium classes is 2.5 students, and between medium and large classes 4 students.	Not specified, but presumably most subjects had been in EMR (special education) classes for at least several months.
MAGI Educational Services, Inc. (1995)	Not specified	Not specified	Not specified	Not specified	Quasi‐experimental observation study	Students and teachers were randomly selected from two class size options: 12:1 and 15:1. Actual number of students observed in the classrooms was generally much lower than the number of students registered.	Formally an increase in some classrooms from 12:1 to 15:1 (so an increase of 3).	Not specified

Author	Type of data (Independent observation/Questionnaire/Other)	Format (Continuous/Categorical/Dichotomous)	Number of measures and timing	Type(s) and name(s) of outcomes (Academic Achievement/Socioemotional outcomes/Wellbeing/Student classroom behaviour (please state name of outcome, e.g. SDQ))	Sample size	Means/regression coefficients/t‐ and F‐statistics/Other	Standard deviations
Metzner (1926)	Pressy first grade reading test was given to those who had done no academic work beyond first grade, and the Stanford Achievement Test for grade 2 and 3 was given to the remaining.	Continuous	Experiment lasted 180 days, tests taken before and at the end.	Reading. Pressy Reading Test and Stanford Achievement Test.	Treated are three classes with 15 students, three classes with 20 students, three classes with 25 students, and three classes with 30 students (total: 270); controls are 12 classes with 22 students (total: 264).	Pre‐test scores and gain scores, Tables 1 and 2.	No SD's reported.
Forness (1985)	The outcome (behaviour) was recorded on each child in specific categories of classroom functioning using an observation system described in detail in Forness, 1983 (available on request from the senior author, i.e. not published).	Percentage of time with a specific behaviour.	Data gathered in April, year not reported.	Behaviour divided into four pre‐determined categories: (a) communication‐ task‐oriented verbal or gestural response (e.g., pupil asks or answers a question, recites or raises hand); (b) attend‐ eye contact to teacher, task materials, or peer who is reciting; (c) not attend‐ eye contact not directed to teacher, task materials, or pupil who is reciting; and (d) disrupt‐behaviour incompatible with on‐task activities (e.g., talks to another pupil when not permitted, speaks out of turn, hits classmates, or throws objects).	26 classes and 393 students. 5 small classes (10–13) with 61 students, 14 medium classes (14–16) with 202 students, and 7 large classes (18–21) with 130 students.	Outcome reported as percentages. The total for each behaviour was computed across all response conditions for each subject. The percentage of time each child received a response from teachers was computed across all types of behaviour as a measure of time the teacher appeared to be involved with each subject, and the same was computed for classmate as a measure of the relative amount of time each subject appeared to interact with peers. Mean percentage by group is reported and overall mean and SD. Table 4.	Table 4
MAGI Educational Services, Inc. (1995)	Two standardised observational instruments were used.	Percentage of time with a specific behaviour.	Not specified	Classroom behaviour observed with: The Code for Instructional Structure and Student Academic Response (MS‐CISSAR), and The Instructional Environment System (TIES II).	753 elementary and secondary students and 203 teachers were randomly selected from the two class size options (12:1 and 15:1).	Percentage of time engaged in different classroom behaviours.	Not reported

**Table 2:** Data extraction: Qualitative studies.

Author(s)	Title	Outlet (journal, dissertation, report)	Year	Type of special education setting (e.g., resource class, special school)	Class size information	Study location	Study design
Gottlieb (1997)	An evaluation Study of the Impact of Modifying Instructional Group Sizes in Resource Rooms and Related Service Groups in New York City	Report	1997	Resource rooms and speech services	In elementary school: 16,26 in 1994‐1995, and 24,39 in 1995‐1996.	New York City, U.S.	Evaluation design using questionnaires, interviews observations, and achievement data.
					In middle school: 20,02 in 1994–1995, and 30,21 in 1995–1996.		
					No accurate data for high schools.		
Huang (2020)	Special education teachers’ perceptions of and practices in individualising instruction for students with intellectual and developmental disabilities in China	Dissertation	2020	Special education schools	Class size ranged from five to 14 students (*M* = 9,4).	Shanghai, China	Interview study
Keith (1993a)	Special Education Program Standards Study. Commonwealth of Virginia. Final Technical Report	Report	1993	Special education classes	Not specified	Virginia, U.S.	Mixed‐methods evaluation study using interviews, observations, document review, and a survey
Prunty (2012)	Voices of students with special educational needs (SEN): views on schooling	British Journal of Learning Support	2012	Special schools or special education classes in mainstream schools	Not specified	Ireland and England	Interview study (focus groups, and individual interviews)
MAGI Educational Services, Inc. (1995)	Results of a Statewide Research Study on the Effects of Class Size in Special Education	Class Size Research Bulletin	1995	Modified Instructional Services (MIS) I classes which covered classes for students who required instructional services in a special class with opportunities for mainstreaming. Students could supposedly spend both full‐time or less in these classes, depending on pull‐out services or involvement with mainstream classrooms.	Class size was increased from 12 to 15 students.	New York, U.S.	The study consisted of a descriptive part and an experimental/observational part. Focus here is on the descriptive study which used a number of complementary data collection methods including document review, public hearings, focus groups, surveys of key informants, and record review.

Author(s)	Theoretical perspective	Research objectives	Student age and gender	Characteristics of student group (e.g., category of special needs, SES)	Teacher education and experience	Parent characteristics (e.g., SES)	Overall quality appraisal
Gottlieb (1997)	Not specified	To evaluate the impact of increased group size on the quality and availability of resource rooms and related service instruction.	Not specified	Not specified	Participants were resource room teachers (representing all levels of schooling), speech therapists, and general education teachers (who had resource room students enroled in their classes).	Not specified	Include for analysis. No philosophical or theoretical perspectives presented and not a lot of information on methods and analytical procedures. However, the paper works well as an evaluation report, and the design chosen is appropriate for an evaluation. The conclusions drawn flow from the descriptive data presented.
Huang (2020)	Critical realism	To investigate and describe Chinese special education teachers’ perceptions and practices related to individualising or adapting instruction for students with intellectual and developmental disabilities.	Grades 1–6	Students with intellectual and developmental disabilities, covering both autism, physical impairments, and intellectual disabilities. Other types of disabilities were less frequently represented.	The participating teachers were Chinese language arts and math special education teachers with an average of 14,6 years of experience teaching students with intellectual and developmental disabilities (range was three‐26 years). All but two participants held Bachelor's degrees as their highest educational level.	Not specified	Include for analysis. Class size is not the main topic of the study, but it is touched upon. The study is well‐performed and clearly reported.
Keith (1993a)	Not specified	To investigate Virginia special education program standards, focusing on local applications of the standards for class size and class mix and the effects of varying class sizes and mix on student outcomes.	Students were from preschool, elementary, middle, and high school. Boys made up 70% of the students in the special education programmes	Students with educable mental retardation, severe emotional disturbance, and specific learning disabilities.	Teachers had worked an average of 6,5 years in their current job, and had worked an an average of 11 years in the field of special education. Almost half the teachers had a Bachelor's degree as their highest educational level, while another 49% held Master's degrees.	Not specified	Exclude from analysis. No philosophical or theoretical perspective stated, very limited description of data collection, and the approach to qualitative analysis is not described. It is unclear in what way the site visits and interview material was used. The paper functions well enough as an evaluation report, but as a qualitative research study, it is inadequately reported and therefore not suited for inclusion.
Prunty (2012)	Perspective of the child	To explore the views of children and young people on their schooling	Not specified	Not clearly presented, but some had physical disabilities, while others had mental disabilities	Not specified	Not specified	Include for analysis. This study is not about differences between different special education settings, but more about differences between mainstream/inclusion and special education. Nonetheless, there are points made here that carry relevance to the issue of special education class size. In terms of methodological quality, the study is well performed and transparently reported.
MAGI Educational Services, Inc. (1995)	Not specified	To examine class size effects on students, service providers, parents, and school districts.	Students were from elementary and secondary grades.	The majority of students were classified as learning disabled.	Not specified	Not specified	Exclude from analysis. No philosophical or theoretical perspective stated, very limited description of data collection, and the approach to qualitative analysis is not described.

#### 5.1.3 Excluded studies

10 studies were initially included, but were later excluded with reasons. A list of these late‐stage excluded studies can be found in Excluded studies, with reasons for exclusion provided.

### 5.2 Risk of bias in included studies

No studies reported on randomised trials. Three studies were assessed using the ROBINS‐I tool: Metzner (1926), Forness (1985), and MAGI Educational Services, Inc. (1995) (the quantitative part of the study). The full risk of bias assessment of the three studies is shown in Table 3. The overall assessment of the three studies resulted in one ‘moderate risk of bias’ assessment (Metzner, 1926) and two ‘critical risk of bias’ assessments (Forness, 1985, and MAGI Educational Services, Inc., 1995). Metzner (1926) was assessed to be a well‐performed study and rated with ‘moderate risk of bias’ across all domains, except for classification bias, which was rated ‘low risk of bias’. Forness, 1985, was assessed as having an overall ‘critical risk of bias’ due to a ‘critical risk of bias’ rating in the confounding domain, after which the rating was stopped. The same was true for MAGI Educational Services, Inc., 1995, which also received a ‘critical risk of bias’ in the confounding domain. In both cases, the reason for judging the confounding domain as ‘critical risk of bias’ was a lack of controls for any confounding factors within the studies.

**Table 3:** Risk of bias assessment of included quantitative studies (ROBINS‐I).

Author	Title	Overall comment	Overall judgement	Confounding bias	Judgement	Selection bias	Judgement	Classification bias	Judgement
Metzner (1926)	Size of class for mentally retarded children	This is a well‐performed study, but unfortunately, SD's are missing and are not possible to retrieve or calculate.	Moderate risk of bias	The authors term it an experiment, but only report that groups of mentally retarded students (four treated and one control) were ‘formed’. Treated are three classes with 15 students, three classes with 20 students, three classes with 25 students, and three classes with 30 students; controls are 12 classes with 22 students. Gender is highly imbalanced, mental age and age in years are reasonably balanced, IQ is reasonably balanced, and SES is not reported (Table 3). Nothing is controlled for.	Moderate risk of bias	8%–10% were replaced due to drop‐out (p. 242), otherwise all children from the initial sample are followed from start to finish. Replacements took the same pre‐tests as students in the initial sample, but the size of the class they attended before they were included as replacements and the timing of the replacement is not reported.	Moderate risk of bias	Nothing of concern	Low risk of bias
Forness (1985)	Effects of class size on attention, communication, and disruption of mildly mentally retarded children	The study is given a rating of critical risk of bias in the confounding domain and the rest is therefore not assessed.	Critical risk of bias	Only age and gender considered (Table 3). There is some imbalance on gender (between small classes vs. medium and large) and a relatively large imbalance on age (between small classes vs. medium and large). All students are characterised as mildly/educable mentally retarded (IQ range of 50–70). Nothing is controlled for.	Critical risk of bias				
MAGI Educational Services, Inc. (1995)	Results of a Statewide Research Study on the Effects of Class Size in Special Education	The study is given a rating of critical risk of bias in the confounding domain and the rest is therefore not assessed.	Critical risk of bias	No confounders considered.	Critical risk of bias				

Author	Deviation bias	Judgement	Missing data	Judgement	Measurement bias	Judgement	Reporting bias	Judgement
Metzner (1926)	Eight percent of the control group and 10% of the treated (not reported separately by the four treatment groups, but reported that the range was 8%–13%) dropped out and were replaced. The size of the class they attended before they were included as replacements and the timing of the replacement is not reported.	Moderate risk of bias	Eight percent of the control group and 10% of the treated (not reported separately by the four treatment groups, but reported that the range was 8%–13%) dropped out and were replaced. Data only shown for those who were in the study by the end of the experiment.	Moderate risk of bias	No mentioning of blinding, otherwise tests are standardised tests (Stanford‐Binet and Pressey reading test)	Moderate risk of bias	No pre‐specified plan of analysis, but otherwise nothing to indicate selective reporting biases	Moderate risk of bias
Forness (1985)								
MAGI Educational Services, Inc. (1995)								

### 5.3 Effects of interventions

#### 5.3.1 Quantitative studies

As noted, two quantitative studies were given a ‘critical risk of bias’ rating corresponding to a risk of bias so high that the findings should not be considered in the data synthesis (Forness, 1985; MAGI Educational Services, Inc., 1995). One study (Metzner, 1926) received a ‘moderate risk of bias’ rating. Unfortunately, Metzner, 1926, did not report SD's and it was not possible to derive them from other values or to retrieve information from the study authors due to the age of the study. Therefore, it was only possible to perform a descriptive data extraction, which is shown in Table 1.

#### 5.3.2 Qualitative studies

Only five qualitative or mixed‐methods studies containing eligible qualitative data (including MAGI Educational Services, Inc., 1995, which was also counted as a quantitative study) were found in the searches. Of these five studies, three were given an overall quality appraisal of ‘Include’ (Gottlieb, 1997; Huang, 2020; Prunty, 2012), whereas two were given an ‘Exclude’ appraisal (Keith, 1993a; MAGI Educational Services, Inc., 1995). With only three eligible studies, two of which contained only limited data specific to special education class size, it was not feasible to perform a thematic synthesis, as we had planned and described in the protocol. Instead, we will present the quality appraisal of the five studies and provide short summaries of the main findings from the three studies that were given an overall appraisal of ‘Include’.

##### Critical appraisal of qualitative studies

Of the five qualitative or mixed methods studies containing eligible qualitative data, three studies were given an overall appraisal of ‘Include’: Gottlieb (1997), Huang (2020) and Prunty (2012). Gottlieb (1997) was found to implement an appropriate design for an evaluation report and reviewers noted that there was a clear link between the conclusions drawn and the descriptive data presented. The study by Huang (2020) was not mainly concerned with class size in special education, but there were a few findings relevant to this review. In terms of methodological quality, the study was well‐performed and transparently reported. Finally, Prunty (2012) mainly explored differences between mainstream/inclusion settings and special education. Nonetheless, there were some findings carrying relevance to special education class size, and as was the case with Huang (2020), the study was transparent and applied a consistent methodological approach.

Two studies were given an overall appraisal of ‘Exclude’, meaning that they were not eligible to be included in a thematic analysis: Keith (1993a), and MAGI Educational Services, Inc. (1995). Both studies were excluded due to a lack of transparency in the reporting of data collection methods and analytical procedures.

The full critical appraisals of qualitative studies can be found in Table 4.

**Table 4:** Quality appraisal of qualitative studies (JBI Critical Appraisal Checklist for Qualitative Research).

Study	Is there congruity between the stated philosophical perspective and the research methodology?	Is there congruity between the research methodology and the research question or objectives?	Is there congruity between the research methodology and the methods used to collect data?	Is there congruity between the research methodology and the representation and analysis of data?	Is there congruity between the research methodology and the interpretation of results?	Is there a statement locating the researcher culturally or theoretically?
Gottlieb (1997)	Although a philosophical perspective is not cited or described, the research design seems appropriate and in line with the evaluative nature of the study aims.	Uses questionnaires, interviews, observations, and achievement data to evaluate changes in resource room group size, in line with the evaluation design.	There is limited information about the questionnaires, interview schedules, and the approach to the qualitative analysis of parental responses. It would have been preferable with a more detailed methodological section.	The authors present data from each source separately. Not much analysis in terms of interpretation or theoretical discussion ‐ descriptive summation only.	The study is purely descriptive.	No theoretical perspectives presented, purely descriptive study.
Huang (2020)	Critical realism is well presented and the author reflects on the congruity between this perspective and the study methodology.	Yes, qualitative interviews are appropriate for exploring the research questions.	Yes, the study methodology and methods are well aligned.	Yes, the analysis reflects the use of qualitative interviews through statements from interviews backing up the analytical points.	Yes, as stated in previous section.	Yes, the study is placed in the Chinese context and within a clear philosophical and methodological tradition.
Keith (1993a)	No philosophical or theoretical perspectives presented.	The research method (mixed methods approach with interviews, survey, and test results) seems appropriate for an evaluative study.	Yes, this is a straightforward evaluation design.	There is very limited description of the collection of qualitative data and the approach to qualitative analysis is not really described.	To some degree, although the study is descriptive and correlational and findings are sometimes phrased as if they were causal in nature.	The study is situated in Virginia. No theoretical or philosophical perspectives presented.
Prunty (2012)	The authors are concerned with the right of children to be heard and the imperative for research to let children's voices be heard**—**and this is reflected in the research methodology where children are active participants in focus groups interviews.	Yes, the research question concerning children's views on schooling is well answered through the use of interviews and focus groups with children.	Yes, since the methodology is centred around child participation and the authors use interview methods designed to elicit the views of children.	Yes, the analysis is centred around statements from children, in line with the research focus on child participation.	Yes, in that children's voices are allowed to take centre‐stage in the analysis and no interpretations are made which are not in sync with the statements made by the participating children.	Yes, children's perspectives and developments towards including and emphasising children's rights are central to the study, with reference to key governance documents and previous research.
MAGI Educational Services, Inc. (1995)	The authors do not state a philosophical perspective and there is hardly any description of the research design and data collection methods.	This is not possible to determine based on the limited information given in the study.	This is not possible to determine based on the limited information given in the study.	This is not possible to determine based on the limited information given in the study.	This is not possible to determine based on the limited information given in the study.	No, apart from information about where the study took place (New York).

Study	Is the influence of the researcher on the research, and vice‐versa, addressed?	Are participants, and their voices, adequately represented?	Is the research ethical according to current criteria or recent studies, and is there evidence of ethical approval by an appropriate body?	Do the conclusions drawn in the research report flow from the analysis, or interpretation, of the data?	Overall appraisal
Gottlieb (1997)	No such considerations made.	This is difficult to say, since this is less of a research study in the traditional sense and more of a descriptive report summing up key points. It is stated that some school leaders/administrative staff/teachers refrained from participation due to insecurity, meaning that the results most likely constitute a best case scenario.	The authors do not touch upon issues of ethics, but mention that some schools refrained from participation due to insecurities (which authors believe may have skewed the results to only show ‘the best case scenario’).	Yes, the conclusions flow from the data, but are only descriptive (not based on data interpretation).	Include for analysis. No philosophical or theoretical perspectives presented and not a lot of information on methods and analytical procedures. However, the paper works well as an evaluation report, and the design chosen is appropriate for an evaluation. The conclusions drawn flow from the descriptive data presented.
Huang (2020)	The researcher reflects on her preconceptions and potential biases.	Yes, through statements from the semi‐structured interviews.	Yes.	Yes, there is a clear connection between the analysis and empirical findings presented and the conclusions made in the study.	Include for analysis. Class size is not the main topic of the study, but it is touched upon. The study is well‐performed and clearly reported.
Keith (1993a)	No.	The description provided of the interview material is too limited to determine this.	The research does not seem unethical, however, n*o* ethical reflections are described.	The conclusions flow from the descriptive data presented, but this is mostly true for the survey data, as it is unclear in what way data from the site visits and interviews was used.	Exclude from analysis. No philosophical or theoretical perspective stated, very limited description of data collection, and the approach to qualitative analysis is not described. It is unclear in what way the site visits and interview material was used. The paper functions well enough as an evaluation report, but as a qualitative research study, it is inadequately reported and therefore not suited for inclusion.
Prunty (2012)	Not discussed, but the authors describe measures taken to make the focus groups/interviews comfortable and safe for the children.	Yes, in line with the focus on making children's voices heard.	Yes, children's voices are valued and authors describe taking measures to make the interviews safe and comfortable for children to participate in.	Yes, the conclusions drawn are clearly founded in the empirical data presented in the analysis.	Include for analysis. This study is not about differences between different special education settings, but more about differences between mainstream/inclusion and special education. Nonetheless, there are points made here that carry relevance to the issue of special education class size. In terms of methodological quality, the study is well performed and transparently reported.
MAGI Educational Services, Inc. (1995)	No.	Some participant statements are presented, but it is not possible to determine whether these statements are representative for the participants as a whole, as there is no information on how the data were analysed (and thus how excerpts from different sources of data were selected).	No ethical considerations, but there is nothing to indicate problems.	Not possible to assess, since it is unclear how data were collected and there is no description of the approach to qualitative analysis.	Exclude from analysis. No philosophical or theoretical perspective stated, very limited description of data collection, and the approach to qualitative analysis is not described.

##### Summary of qualitative findings

In the following, the three qualitative or mixed methods studies given an overall appraisal of ‘Include’ are individually summarised with a focus on findings of relevance to special education class size.

Gottlieb (1997) explored the impact of increases in instructional group size in resource rooms in the New York City Public Schools by examining increases in 45 public elementary, middle, and senior high schools. The empirical data were gathered through both qualitative and quantitative data collection methods. Teachers, administrators and principals were interviewed alongside 31 h of observations in resource rooms. Furthermore, questionnaires were distributed to parents and analyses of standardised reading and arithmetic achievement data were performed.

Findings indicated that the increases in instructional group size economically saved the resource room program around 26 million dollars. However, there was a substantial decrease in the reading achievement scores of resource room students, especially at the sixth grade level. Math scores also declined, but not significantly. Furthermore, interviews with resource room teachers suggested that the increase in instructional group size reduced teachers' ability to help students. This was in line with the independent observations which revealed that teachers spent very little time on individual instruction and more time on group instruction and accompanying students to and from their classrooms.

Finally, 25 school principals were interviewed and the conclusion drawn from these interviews was ‘…that principals did not think increases in the instructional group size was a good idea’; in fact, one principal was quoted for saying: ‘You don't have to be a rocket scientist to know this (increased instructional group size in resource rooms) was a bad idea’ (Gottlieb, 1997, p.20). Based on the findings of the study, authors made the recommendation that no more than five students should receive resource room instruction at one time.

Huang (2020) aimed to investigate Chinese special education teachers' perceptions and practices related to individualising or adapting instruction for students with intellectual and developmental disabilities (IDD). Specifically, the investigation focused on teachers who taught elementary Chinese language arts and math in public special education schools for students with IDD in Shanghai. A qualitative research design based on in‐depth semi‐structured interviews with 31 teachers from six schools was utilised. Teachers reported using strategies of dividing students into smaller groups within the classroom based on the students' intellectual abilities to provide students with individualised instruction. Even though the teachers wanted to address student differences, they admitted that it was difficult to provide adaptations to fully meet the students' individual needs and described specific challenges and barriers associated with this. Here, more than half of the participants emphasised that school contextual factors such as large class size and/or insufficient personnel helping out in the classrooms had an influence on teachers' ability to pay attention to the individual needs of students. Therefore, many participants pointed out that having one or more teaching assistants or smaller classes would be helpful.

Prunty (2012) mostly explored the perspectives of students with special needs on segregated special education versus mainstreamed/inclusive settings in Ireland and England. The empirical material was gathered through six focus group interviews and four individual interviews with children and young people with special educational needs. Some of these children had experiences from both mainstream and special education settings. Findings suggested that many students preferred segregated placement because of smaller classes and easier access to one‐on‐one instruction with teachers. Especially literacy support and diverse teaching styles for math were valued among the students. As an example, one student gave the following reason for preferring special class over mainstream placement: ‘… more adult help and smaller classes and stuff’ (Prunty, 2012, p. 30).

## 6 DISCUSSION

### 6.1 Summary of main results

The major finding of the present review is that there are very few contemporary studies exploring the effects of small class sizes in special education. It was not possible to conduct a meta‐analysis nor a thematic qualitative synthesis from the studies found in this review, despite the breadth and comprehensiveness of the search strategy. It follows that there is no basis for broader interpretations regarding the effects of small class sizes in special education based on the studies located in this review. However, findings from the included qualitative studies show that smaller class sizes in special education are the most preferred option among the students, teachers, and school principals participating in these studies due to the possibilities afforded in terms of providing individualised and targeted instruction to each student.

### 6.2 Overall completeness and applicability of evidence

We performed a comprehensive electronic database search, combined with extensive grey literature searches, hand‐searches of key journals, and citation‐tracking. All references were screened by two independent screeners from the review team (JER, MHC, MWK), and at least one review author (AB, TF, NTD) assessed all included studies against inclusion criteria.

We believe that all publicly available quantitative studies on the effects of small class sizes in special education up to the censor date were identified during the review process. As can be seen from the included qualitative studies, class size was not the sole research focus of the studies; in fact, two of the included studies (Huang, 2020; Prunty, 2012) presented findings of relevance to the present review, despite the fact that the research objectives in these studies did not specifically target class size issues. It is possible that there are other qualitative studies where findings may be of relevance to the present review which we have not managed to locate despite our comprehensive search efforts.

23 references were not obtained in full text and one study provided insufficient information to permit us to calculate an effect size.

### 6.3 Quality of the evidence

Three studies containing eligible quantitative data were assessed using the ROBINS‐I tool. As a result, two studies were given a ‘critical risk of bias’ rating (Forness, 1985, and MAGI Educational Services, Inc., 1995). One study (Metzner, 1926) received a ‘moderate risk of bias’ rating; unfortunately, this study did not report SD's and it was not possible to derive them from other values or to retrieve information from the study authors.

Five studies containing eligible qualitative data were rated using an adapted version of the JBI Critical Appraisal Checklist for Qualitative Research (Joanna Briggs Institute, 2017; Lockwood, 2015). Of these five studies, three were assessed to be of sufficient quality and two were assessed to be of insufficient quality due to lack of transparency and methodological clarity.

### 6.4 Potential biases in the review process

We are unable to comment on the possibility of publication bias as no meta‐analysis could be conducted. Thus, we cannot rule out that there are still some missing studies.

We believe that there are no other potential biases in the review process as two members of the review team independently coded the included studies. Any disagreements were resolved by discussion. Further, decisions about inclusion of studies were made by two members of the review team and one review author. Assessment of study quality and numeric data extraction was made by the review authors (AB, TF, NTD) and checked by a second review author.

### 6.5 Agreements and disagreements with other studies or reviews

As noted in the background section, few authors have previously tried to review the available literature on special education class sizes, and these reviews have not followed rigorous, systematic frameworks, such as those applied in the current review. Previous studies have pointed to the lack of evidence surrounding special education class size, but it was our hope that by applying extensive, systematic literature searches that were up‐to‐date with the latest developments in special education, we would reach a conclusion extending further than simply a call for more research. Nonetheless, this is exactly where we are left: calling for more research and hoping that the coming years will bring an increased interest in special education to the benefit of students, teachers, administrators, parents, and systematic reviewers alike.

## 7 AUTHORS' CONCLUSIONS

### 7.1 Implications for practice

The research literature to this day provides little guidance on what the optimal class size is for students with special educational needs in segregated special education settings. Only three studies, published between 1926 and 1995, contained eligible quantitative data and were included in the review. Following assessment with the ROBINS‐I tool, two of these studies were given a ‘critical risk of bias’ rating; the last study was given ‘a moderate risk of bias’ rating, but no standard deviations could be derived. Therefore, it was not possible to perform meta‐analysis. Findings from the review of qualitative studies were also limited; out of five studies, three were assessed to be of sufficient methodological quality and were individually summarised, since it was not feasible to perform a thematic synthesis.

Until further research evidence is available, decision‐makers, parents, and teachers are best guided by relying on individual assessments of children and local best practice experiences in determining the optimal class size arrangements for different groups of children with special educational needs. As with all educational interventions, the effects of different class sizes in special education will likely be influenced by a host of contextual factors linked to the workings of different local and national educational systems. Adding to this contextual diversity is the fact that special needs provision, even within local contexts, is of a varied and specialised nature, often encompassing multiple types of provision for children and young people with very diverse special educational needs. What is evident is therefore that designing high‐quality special education classroom environments is a task that requires specialist knowledge about different types of special educational needs, insight into local types of school provision, and the ability to observe individual children and take their needs into consideration.

### 7.2 Implications for research

Findings from the present review suggest that there is an urgent need for more research on the effects of different class sizes in segregated special education using robust estimation techniques to, as far as possible, isolate the class size effect. From both a practical and an ethical standpoint, performing randomised trials within this area of research would likely not be feasible. However, a possible route would be to exploit the opportunities afforded by natural experiments where alterations to special education class sizes occur due to, for example, policy changes. Furthermore, there is also a need for more qualitative research on the way in which students, teachers, and parents experience different class sizes in special education, as they are the ones whose lives are most directly affected by the conditions surrounding different special education provisions. Such research could also look into the interplay between class size and other structural conditions (such as student–teacher ratio). Future qualitative research should be particularly concerned with providing a safe place for children and young people with special needs to voice their perspectives since it is the right of every child to be involved in decisions concerning his or her life and wellbeing. This imperative is reflected in the following statement from Prunty, 2012: ‘As important decisions are being made with regard to legislation, policy and practice on educational provision for students with special educational needs, it is crucial that the views of the key players, the children, continue to be heard and considered’ (Prunty, 2012, p. 29‐30).

## CONTRIBUTIONS OF AUTHORS

Content: Anja Bondebjerg, Nina Thorup Dalgaard

Systematic review methods: Trine Filges, Anja Bondebjerg, Nina Thorup Dalgaard

Statistical analysis: Trine Filges

Information retrieval: Bjørn Christian Arleth Viinholt (information specialist)

## DECLARATIONS OF INTEREST

None of the review authors or assistants have conflicts of interest related to this review.

## DIFFERENCES BETWEEN PROTOCOL AND REVIEW

We stated in the protocol that we would search Open Grey (now Dans Easy). However, we chose to refrain from searching this resource on advice from information specialist Elizabeth Bengtsen (VIVE), who informed us that Open Grey/Dans Easy contains a lot of items that researchers and students can import by themselves without formal quality control. This is opposed to resources such as EBSCO Open Dissertations which contain only approved dissertations. We therefore chose to remove Open Grey/Dans Easy from our list of references to avoid unneccesary ‘noise’, focusing instead on resources with a higher degree of quality control.

Furthermore, we had planned to perform grey literature searches on the website of The European Educational Research Association (EERA). However, this website turned out to be very limited in terms of search functions, which is why we chose to perform separate hand‐searches in EERA's journal, European Educational Research Journal, instead. These searches are documented alongside the other hand‐searches in Supporting Information: Appendix 4.

In the protocol, we stated that we would perform searches in ProQuest Dissertations & Theses Global (ProQuest), but we were unable to do so due to lack of access. Nonetheless, we believe that our other searches were comprehensive enough to secure adequate coverage of dissertations (which are also included in several of the other databases and grey literature resources included in the search).

We planned to conduct a data synthesis using standard techniques for meta‐analytic reviews. There were, however, no studies to be included in a meta‐analysis and therefore no studies for moderator analysis to be performed and we were unable to comment on the possibility of publication bias. Similarly, due to the limited number of qualitative studies, we did not conduct a thematic synthesis of findings, but chose to summarise findings from each study separately.

## PUBLISHED NOTES

**Table:** 

Characteristics of excluded studies	
Bloom (1992)	
**Reason for exclusion**	Compares students from one district to students in another district (unit bias)
Dykstra (2013)	
**Reason for exclusion**	This study investigates issues related to instructional group size, not class size.
Furno (1967)	
**Reason for exclusion**	Class size is measured as: the pupil's median class size over a period of 4 years, or, in particular, the school years 1959–1960, 1960–1961, 1961–1962, and 1962–1963. Only outcomes averaged over the six school years:1959–1960, 1960–1961, 1961–1962, 1962–1963, 1963–1964, and 1964–1965 are analysed.
Hart (2011)	
**Reason for exclusion**	Not about class size: A total of 33 children with ADHD were randomly assigned within days to either small‐group instruction, whole‐group instruction, or independent seatwork. The effects of instructional contexts on on‐task behaviour during instruction and on‐task behaviour and work productivity during testing were examined.
Keith (1993b)	
**Reason for exclusion**	No numbers reported.
Patterson (2016)	
**Reason for exclusion**	Compares self‐contained classrooms to inclusion and mainstream. Also has a class size component specifically by placement type, but there is no variation in self‐contained classroom sizes (only 1–10), see Table 6.
Snart (1985)	
**Reason for exclusion**	Investigates student/teacher ratio, not class size. Also, the outcome in this study is not a validated measure of student classroom behaviour. Furthermore, authors state the following on p. 293: ‘Limited research access to the classrooms discussed within this study resulted in a confounding of condition with classroom, since we had agreed to spend only one full day per classroom’ (unit bias).
Steinbrenner (2015)	
**Reason for exclusion**	The classrooms analysed served between six and ten students (i.e., a varying number), but the analysis is not about class size, but instructional group size.
	Large groups is the whole class: The classrooms all used some large group instruction (e.g., morning group, academic instruction).
	Small group: A few of the classrooms also had small group times, in which the classroom staff worked with dyads or triads on academic tasks such as worksheets or book reading. The observations were planned to be conducted during two one‐to‐one sessions, two small group sessions (i.e., 2–3 students) and two large group sessions (i.e., 4 or more students) when possible. However, many classrooms did not have regularly scheduled small group sessions; therefore, additional large group sessions were observed for students who did not participate in small group sessions
Thurlow (1988)	
**Reason for exclusion**	Does not investigate class size, but how many teachers pr. student in instructional group, where the same students can be in more than one grouping.
Thurlow (1993)	
**Reason for exclusion**	Does not investigate class size, but how many teachers pr. student in instructional group, where the same students can be in more than one grouping (see p. 310 and Table 2).

## SOURCES OF SUPPORT

**Internal sources**

- VIVE, The Danish Centre for Social Science Research, Denmark

**External sources**

- No sources of support provided

## Supporting information

Supporting information.

## Footnotes

^1^
For more information on the IDEA Act disability categories, go to: https://sites.ed.gov/idea/regs/b/a/300.8 (the U.S. Department of Education's Individuals with Disabilities Education Act (IDEA) website)
