# Supplementary material for: The effects of small class sizes on students' academic achievement, socioemotional development and well‐being in special education: A systematic review
Source: Campbell Syst Rev. 2023 Jul 14;19(3):e1345. doi: 10.1002/cl2.1345 (PMC10346380; doi:10.1002/cl2.1345)
Supplement: Supplementary file 1 — Supporting information. [file CL2-19-e1345-s001.docx]

# Appendices

## 1 Justification of exclusion of studies using an instrumental variable (IV) approach

Studies using instrument variables (IV) for causal inference in non-randomised studies were not included as the interpretation of IV estimates is challenging. IV only provides an estimate for a specific group, namely people whose behaviour changes due to changes in the particular instrument used. It is not informative about effects on never-takers and always-takers because the instrument does not affect their treatment status. The estimated effect is thus applicable only to the subpopulation whose treatment status is affected by the instrument. As a consequence, the effects differ for different IVs and care has to be taken as to whether they provide useful information. The effect is interesting when the instrument it is based on is interesting in the sense that it corresponds to a policy instrument of interest. Further, if those that are affected by the instrument are not affected in the same way, the IV estimate is an average of the impacts of changing treatment status in both directions, and cannot be interpreted as a treatment effect. To turn the IV estimate into a LATE requires a monotonicity assumption. The movements induced by the instrument go in one direction only, from no treatment to treatment. The IV estimate, interpreted as a LATE, is only applicable to the complier population, those that are affected by the instrument in the ‘right way’. It is not possible to characterise the complier population as an observation’s subpopulation cannot be determined and defiers do not exist by assumption.

In the binary-treatment–binary-instrument context, the IV estimate can, given monotonicity, be interpreted as a LATE; i.e. the average treatment effect for the subpopulation of compliers. If treatment or instruments are not binary, interpretation becomes more complicated. In the binary-treatment–multivalued-instrument (ordered to take values from 0 to *J*) context, the IV estimate, given monotonicity, is a weighted average of pairwise LATE parameters (comparing subgroup *j* with subgroup *j*−1). The IV estimate can thus be interpreted as the weighted average of average treatment effects in each of the *J* subgroups of compliers. In the multivalued-treatment (ordered to take values from 0 to *T*) – multivalued-instrument (ordered to take values from 0 to *J*) context, the IV estimate for *each pair of instrument values*, given monotonicity, is a weighted average of the effects from going from *t*-1 to *t* for persons induced by the change in the value of the instrument to move from any level below *t* to the level *t* or any level above. Persons can be counted multiple times in forming the weights.

## 2 Screening guide

First level screening was performed on the basis of titles and abstracts. Second level was on the basis of full texts.

The below screening questions and guidance texts were used to guide the screening process. A study was excluded if one or more of the answers to questions 1-4 were ‘No’. If the answers to questions 1 to 4 were ‘Yes’ or ‘Uncertain’, the full text of the study was retrieved to assess second level eligibility.

**Screening questions:**

*1. Does the study measure the effects of special education (may be referred to as e.g. segregated placement, special class, self-contained special education classes, or resource rooms)?*

Yes - include

No – stop here and exclude

Uncertain - include

Question 1 guidance:

Special education refers to educational settings catering exclusively to children with special educational needs, i.e. groups or classes contain only special education students. Placement in a special education setting may be full time or part time.

*2. Does the study measure effects for students with special needs?*

Yes - include

No – stop here and exclude

Uncertain – include

Question 2 guidance:

The population of this review are children with special educational needs in grades K to 12 (or the equivalent in European countries) in special education. Studies that meet inclusion criteria will be accepted from all countries. In this review we apply the widely used definition from the US Individuals with Disabilities Education Act (IDEA), in which special needs are divided into 13 different disability categories under which children are eligible for services. These categories are:

· specific learning disability (covers challenges related to a child’s ability to read, write, listen, speak or do math, e.g. dyslexia or dyscalculia),

· other health impairment (covers conditions limiting a child’s strength, energy, or alertness, e.g. ADHD),

· autism spectrum disorder (ASD),

· emotional disturbance (may include e.g. anxiety, obsessive-compulsive disorder and depression),

· speech or language impairment (covers difficulties with speech or language, e.g. language problems affecting a child’s ability to understand words or express herself),

· visual impairment (covers eyesight problems, including partial sight and blindness),

· deafness (covers instances where a child cannot hear most or all sounds, even with a hearing aid),

· hearing impairment (refers to a hearing loss not covered by the definition of deafness),

· deaf-blindness (covers children suffering from both severe hearing and vision loss),

· orthopaedic impairment (covers instances when a child has problems with bodily function or ability, as in the case of cerebral palsy),

· intellectual disability (covers below-average intellectual ability),

· traumatic brain injury (covers brain injuries caused by accidents or other kinds of physical force),

· multiple disabilities (children with more than one condition covered by the IDEA criteria).

Note that the above categories should not be seen as exhaustive, but as guiding tools. Other definitions of special needs than the above mentioned will also be eligible. If in doubt, include study for full-text screening.

*3. Is the report/article a quantitative study with a comparison condition, or a qualitative study collecting empirical data?*

Yes - include

No – stop here and exclude

Uncertain – include

Question 3 guidance:

Quantitative studies: We are only interested in primary quantitative studies with a control or comparison group. Eligible study designs are: Randomized controlled trials (RCTs), Quasi-randomized controlled trial designs (QRCTs), Quasi-experimental studies (QES), and repeated-measures experimental designs in which the same caregiver and/or children are observed under different conditions within a short time span. Studies reporting associations in cohort, cross-sectional and longitudinal study designs without a comparison group are not eligible.

Qualitative studies: We will include all types of empirical qualitative studies that collect primary data and provide descriptions of main methodological issues such as sampling, data collection procedures and type of data analysis. A qualitative study may apply a wealth of data collection methods, with participant observation, in-depth interviews, or focus groups being examples of possible methods we may encounter in the included studies.

Note: We are not interested in theoretical papers on the topic, or surveys/reviews of studies of the topic. (This question may be difficult to answer on the base of titles and abstracts alone). If in doubt, include for second level screening on full text.

## 3 Database searches with search results

**ERIC** (1966-2021). Search performed 22/04/2021. Interface - EBSCOhost Research Databases. Search Screen - Advanced Search Search modes - Boolean/Phrase.

| **Search** | **Search Terms** | **Results** |
| --- | --- | --- |
| S17 | S4 AND S9 AND S16 | 2,063 |
| S16 | S10 OR S11 OR S12 OR S13 OR S14 OR S15 | 947,908 |
| S15 | DE (“Qualitative Research” OR “Ethnography” OR “Case Studies” OR “Evaluation Methods” OR “Field Studies” OR “Focus Groups” OR “Interviews” OR “Mixed Methods Research” OR “Naturalistic Observation” OR “Participant Observation” OR “Classroom Observation Techniques” OR “Observation” OR “Action Research”) | 216,863 |
| S14 | AB (qualitative* OR ethnograp* OR “case stud*” OR evaluation* OR “focus group*” OR interview* OR “mixed method*” OR observation*) | 350,901 |
| S13 | TI (qualitative* OR ethnograp* OR “case stud*” OR evaluation* OR “focus group*” OR interview* OR “mixed method*” OR observation*) | 81,215 |
| S12 | DE ("Effect Size" OR "Control Groups" OR "Experimental Groups" OR "Experiments" OR "Matched Groups" OR "Quasiexperimental Design" OR "Randomized Controlled Trials" OR "Comparative Testing" OR "Intervention") | 73,126 |
| S11 | AB (effect* OR trial* OR experiment* OR "control group*" OR random* OR impact* OR compar* OR difference*) | 650,198 |
| S10 | TI (effect* OR trial* OR experiment* OR "control group*" OR random* OR impact* OR compar* OR difference*) | 163,721 |
| S9 | S5 OR S6 OR S7 OR S8 | 30,419 |
| S8 | DE ("Class Size" OR "Small Classes" OR "Teacher Student Ratio") | 4,640 |
| S7 | TX (group* OR class*) N5 (size*) | 9,114 |
| S6 | AB (group* OR class*) AND AB (size* OR ratio*) | 27,738 |
| S5 | TI (group* OR class*) AND TI (size* OR ratio*) | 1,037 |
| S4 | S1 OR S2 OR S3 | 117,763 |
| S3 | DE ("Special Needs Students" OR “Special Schools" OR "Residential Schools" OR “Educationally Disadvantaged” OR “Developmental Delays” OR "Students with Disabilities" OR "Special Classes" OR "Special Education" OR "Self Contained Classrooms" OR "Resource Room") | 42,113 |
| S2 | AB (special*) AND AB (need* OR education OR child* OR student* OR pupil*) | 95,514 |
| S1 | TI (special*) AND TI (need* OR education OR child* OR student* OR pupil*) | 15,826 |

**Academic Search Premier** (1931-2021). Search performed 22/04/2021. Interface - EBSCOhost Research Databases. Search Screen - Advanced Search Search modes - Boolean/Phrase.

| **#** | **Query** | **Results** |
| --- | --- | --- |
| S17 | S4 AND S9 AND S16 | 4,923 |
| S16 | S10 OR S11 OR S12 OR S13 OR S14 OR S15 | 13,381,604 |
| S15 | DE (“Qualitative Research” OR “Case Studies” OR “EVALUATION methodology” OR “Focus Groups” OR “Mixed Methods Research” OR “Participant Observatio” OR “OBSERVATION (Educational method)” OR “Action Research”) | 143,166 |
| S14 | AB (qualitative* OR ethnograp* OR “case stud*” OR evaluation* OR “focus group*” OR interview* OR “mixed method*” OR observation*) | 2,401,114 |
| S13 | TI (qualitative* OR ethnograp* OR “case stud*” OR evaluation* OR “focus group*” OR interview* OR “mixed method*” OR observation*) | 597,740 |
| S12 | DE ("Effect Size" OR "Control Groups" OR "Experimental Groups" OR "Experiments" OR "Matched Groups" OR "Randomized Controlled Trials") | 126,017 |
| S11 | AB (effect* OR trial* OR experiment* OR "control group*" OR random* OR impact* OR compar* OR difference*) | 11,544,632 |
| S10 | TI (effect* OR trial* OR experiment* OR "control group*" OR random* OR impact* OR compar* OR difference*) | 2,660,524 |
| S9 | S5 OR S6 OR S7 OR S8 | 659,929 |
| S8 | DE ("Class Size" OR "GROUP size") | 2,571 |
| S7 | TX (group* OR class*) N5 (size*) | 232,549 |
| S6 | AB (group* OR class*) AND AB (size* OR ratio*) | 478,817 |
| S5 | TI (group* OR class*) AND TI (size* OR ratio*) | 4,392 |
| S4 | S1 OR S2 OR S3 | 187,492 |
| S3 | DE ("Special Needs Students" OR “EDUCATIONALLY disadvantaged students” OR “DEVELOPMENTAL disabilities” OR "SPECIAL classes (Education)" OR "Special Education" OR "SELF-Contained Classrooms" OR "Resource Room") | 33,928 |
| S2 | AB (special*) AND AB (need* OR education OR child* OR student* OR pupil*) | 162,348 |
| S1 | TI (special*) AND TI (need* OR education OR child* OR student* OR pupil*) | 12,941 |

**EconLit** (1969–2021). Search performed 22/04/2021. Interface - EBSCOhost Research Databases. Search Screen - Advanced Search Search modes - Boolean/Phrase.

| **Search** | **Search Terms** | **Results** |
| --- | --- | --- |
| S13 | S3 AND S7 AND S12 | 86 |
| S12 | S8 OR S9 OR S10 OR S11 | 651,129 |
| S11 | AB (qualitative* OR ethnograp* OR “case stud*” OR evaluation* OR “focus group*” OR interview* OR “mixed method*” OR observation*) | 84,506 |
| S10 | TI (qualitative* OR ethnograp* OR “case stud*” OR evaluation* OR “focus group*” OR interview* OR “mixed method*” OR observation*) | 31,162 |
| S9 | AB (effect* OR trial* OR experiment* OR "control group*" OR random* OR impact* OR compar* OR difference*) | 529,118 |
| S8 | TI (effect* OR trial* OR experiment* OR "control group*" OR random* OR impact* OR compar* OR difference*) | 177,206 |
| S7 | S4 OR S5 OR S6 | 15,554 |
| S6 | TX (group* OR class*) N5 (size*) | 2,669 |
| S5 | AB (group* OR class*) AND AB (size* OR ratio*) | 15,281 |
| S4 | TI (group* OR class*) AND TI (size* OR ratio*) | 563 |
| S3 | S1 OR S2 | 5,727 |
| S2 | AB (special*) AND AB (need* OR education OR child* OR student* OR pupil*) | 5,591 |
| S1 | TI (special*) AND TI (need* OR education OR child* OR student* OR pupil*) | 223 |

**APA PsycINFO** (1890–2021). Search performed 22/04/2021. Interface - EBSCOhost Research Databases. Search Screen - Advanced Search Search modes - Boolean/Phrase.

| **#** | **Query** | **Results** |
| --- | --- | --- |
| S17 | S4 AND S9 AND S16 | 2,080 |
| S16 | S10 OR S11 OR S12 OR S13 OR S14 OR S15 | 3,079,078 |
| S15 | DE (“Qualitative Methods” OR “Ethnography” OR “Qualitative Measures” OR “Interviews” OR “Mixed Methods Research” OR “Observation Methods” OR “Participant Observation” OR “Action Research”) | 37,220 |
| S14 | AB (qualitative* OR ethnograp* OR “case stud*” OR evaluation* OR “focus group*” OR interview* OR “mixed method*” OR observation*) | 812,464 |
| S13 | TI (qualitative* OR ethnograp* OR “case stud*” OR evaluation* OR “focus group*” OR interview* OR “mixed method*” OR observation*) | 168,537 |
| S12 | DE ("Effect Size (Statistical)" OR "Experiment Controls" OR "Randomized Controlled Trials" OR "School Based Intervention" OR "Intervention") | 91,826 |
| S11 | AB (effect* OR trial* OR experiment* OR "control group*" OR random* OR impact* OR compar* OR difference*) | 2,527,513 |
| S10 | TI (effect* OR trial* OR experiment* OR "control group*" OR random* OR impact* OR compar* OR difference*) | 677,643 |
| S9 | S5 OR S6 OR S7 OR S8 | 92,418 |
| S8 | DE ("Class Size") | 323 |
| S7 | TX (group* OR class*) N5 (size*) | 14,896 |
| S6 | AB (group* OR class*) AND AB (size* OR ratio*) | 90,640 |
| S5 | TI (group* OR class*) AND TI (size* OR ratio*) | 1,685 |
| S4 | S1 OR S2 OR S3 | 133,630 |
| S3 | DE ("Special Needs" OR "Special Education" OR "Special Education Students" OR “Developmental Disabilities” OR "Students with Disabilities") | 50,411 |
| S2 | AB (special*) AND AB (need* OR education OR child* OR student* OR pupil*) | 98,791 |
| S1 | TI (special*) AND TI (need* OR education OR child* OR student* OR pupil*) | 11,539 |

**SocINDEX** (1895–2021). Search performed 22/04/2021. Interface - EBSCOhost Research Databases. Search Screen - Advanced Search Search modes - Boolean/Phrase.

| **Search** | **Search Terms** | **Results** |
| --- | --- | --- |
| S17 | S4 AND S9 AND S16 | 871 |
| S16 | S10 OR S11 OR S12 OR S13 OR S14 OR S15 | 931,636 |
| S15 | DE (“QUALITATIVE research” OR “INTERVIEWING” OR “Participant Observation” OR “Action Research”) | 33,695 |
| S14 | AB (qualitative* OR ethnograp* OR “case stud*” OR evaluation* OR “focus group*” OR interview* OR “mixed method*” OR observation*) | 281,859 |
| S13 | TI (qualitative* OR ethnograp* OR “case stud*” OR evaluation* OR “focus group*” OR interview* OR “mixed method*” OR observation*) | 59,565 |
| S12 | DE ("SOCIAL science experiments" OR "Randomized Controlled Trials" OR "CLINICAL trials" OR "EARLY intervention (Education)" OR "EDUCATIONAL intervention") | 6,233 |
| S11 | AB (effect* OR trial* OR experiment* OR "control group*" OR random* OR impact* OR compar* OR difference*) | 716,005 |
| S10 | TI (effect* OR trial* OR experiment* OR "control group*" OR random* OR impact* OR compar* OR difference*) | 163,733 |
| S9 | S5 OR S6 OR S7 OR S8 | 49,645 |
| S8 | DE ("Class Size" OR "GROUP size" OR "INCLUSIVE education" OR "MAINSTREAMING in special education") | 1,175 |
| S7 | TX (group* OR class*) N5 (size*) | 31,815 |
| S6 | AB (group* OR class*) AND AB (size* OR ratio*) | 21,193 |
| S5 | TI (group* OR class*) AND TI (size* OR ratio*) | 582 |
| S4 | S1 OR S2 OR S3 | 38,543 |
| S3 | DE ("CHILDREN with disabilities" OR "Special Education" OR “Developmental Disabilities” OR "DEVELOPMENTALLY disabled children" OR "DEVELOPMENTALLY disabled youth") | 6,477 |
| S2 | AB (special*) AND AB (need* OR education OR child* OR student* OR pupil*) | 33,365 |
| S1 | TI (special*) AND TI (need* OR education OR child* OR student* OR pupil*) | 2,437 |

**International Bibliography of the Social Sciences** (**IBSS**, 1951–2021). Search performed 23/04/2021. Search performed through the ProQuest advanced search interface.

| **Search** | **Search Terms** | **Results** |
| --- | --- | --- |
| S18 | [S4 AND S10 AND S17](https://search.proquest.com/recentsearches.recentsearchtabview.recentsearchesgridview.scrolledrecentsearchlist.checkdbssearchlink:rerunsearch/B9825B90ED934565PQ/None?site=ibss&t:ac=RecentSearches) | [500](https://search.proquest.com/recentsearches.recentsearchtabview.recentsearchesgridview.scrolledrecentsearchlist.checkdbssearchlink_0:rerunsearch/B9825B90ED934565PQ/None?site=ibss&t:ac=RecentSearches) |
| S17 | [S11 OR S12 OR S13 OR S14 OR S15 OR S16](https://search.proquest.com/recentsearches.recentsearchtabview.recentsearchesgridview.scrolledrecentsearchlist.checkdbssearchlink:rerunsearch/653C519FBA8B48CAPQ/None?site=ibss&t:ac=RecentSearches) | [1,335,128](https://search.proquest.com/recentsearches.recentsearchtabview.recentsearchesgridview.scrolledrecentsearchlist.checkdbssearchlink_0:rerunsearch/653C519FBA8B48CAPQ/None?site=ibss&t:ac=RecentSearches) |
| S16 | [MAINSUBJECT.EXACT(" Qualitative studies") OR MAINSUBJECT.EXACT(" Qualitative research") OR MAINSUBJECT.EXACT("Ethnography") OR MAINSUBJECT.EXACT("case studies") OR MAINSUBJECT.EXACT("interviews") OR MAINSUBJECT.EXACT("focus groups") OR MAINSUBJECT.EXACT("Classroom observation")](https://search.proquest.com/recentsearches.recentsearchtabview.recentsearchesgridview.scrolledrecentsearchlist.checkdbssearchlink:rerunsearch/325A5A1707FC4EA9PQ/None?site=ibss&t:ac=RecentSearches) | [106,680](https://search.proquest.com/recentsearches.recentsearchtabview.recentsearchesgridview.scrolledrecentsearchlist.checkdbssearchlink_0:rerunsearch/325A5A1707FC4EA9PQ/None?site=ibss&t:ac=RecentSearches) |
| S15 | [AB(qualitative* OR ethnograp* OR “case stud*” OR evaluation* OR “focus group*” OR interview* OR “mixed method*” OR observation*)](https://search.proquest.com/recentsearches.recentsearchtabview.recentsearchesgridview.scrolledrecentsearchlist.checkdbssearchlink:rerunsearch/BD323C6F0DFC4544PQ/None?site=ibss&t:ac=RecentSearches) | [324,056](https://search.proquest.com/recentsearches.recentsearchtabview.recentsearchesgridview.scrolledrecentsearchlist.checkdbssearchlink_0:rerunsearch/BD323C6F0DFC4544PQ/None?site=ibss&t:ac=RecentSearches) |
| S14 | [TI(qualitative* OR ethnograp* OR “case stud*” OR evaluation* OR “focus group*” OR interview* OR “mixed method*” OR observation*)](https://search.proquest.com/recentsearches.recentsearchtabview.recentsearchesgridview.scrolledrecentsearchlist.checkdbssearchlink:rerunsearch/E9BF0FBFF17C4090PQ/None?site=ibss&t:ac=RecentSearches) | [99,551](https://search.proquest.com/recentsearches.recentsearchtabview.recentsearchesgridview.scrolledrecentsearchlist.checkdbssearchlink_0:rerunsearch/E9BF0FBFF17C4090PQ/None?site=ibss&t:ac=RecentSearches) |
| S13 | [MAINSUBJECT.EXACT("Effectiveness") OR MAINSUBJECT.EXACT("Efectiveness studies") OR MAINSUBJECT.EXACT("Effects") OR MAINSUBJECT.EXACT("Experiments") OR MAINSUBJECT.EXACT("Randomized controlled trials") OR MAINSUBJECT.EXACT("Intervention") OR MAINSUBJECT.EXACT("Early Intervention")](https://search.proquest.com/recentsearches.recentsearchtabview.recentsearchesgridview.scrolledrecentsearchlist.checkdbssearchlink:rerunsearch/5F738658214D428BPQ/None?site=ibss&t:ac=RecentSearches) | [45,186](https://search.proquest.com/recentsearches.recentsearchtabview.recentsearchesgridview.scrolledrecentsearchlist.checkdbssearchlink_0:rerunsearch/5F738658214D428BPQ/None?site=ibss&t:ac=RecentSearches) |
| S12 | [AB(effect* OR trial* OR experiment* OR "control group*" OR random* OR impact* OR compar* OR difference*)](https://search.proquest.com/recentsearches.recentsearchtabview.recentsearchesgridview.scrolledrecentsearchlist.checkdbssearchlink:rerunsearch/1E30B26A6BB64C2APQ/None?site=ibss&t:ac=RecentSearches) | [920,344](https://search.proquest.com/recentsearches.recentsearchtabview.recentsearchesgridview.scrolledrecentsearchlist.checkdbssearchlink_0:rerunsearch/1E30B26A6BB64C2APQ/None?site=ibss&t:ac=RecentSearches) |
| S11 | [TI(effect* OR trial* OR experiment* OR "control group*" OR random* OR impact* OR compar* OR difference*)](https://search.proquest.com/recentsearches.recentsearchtabview.recentsearchesgridview.scrolledrecentsearchlist.checkdbssearchlink:rerunsearch/ED12066CDCF64031PQ/None?site=ibss&t:ac=RecentSearches) | [307,397](https://search.proquest.com/recentsearches.recentsearchtabview.recentsearchesgridview.scrolledrecentsearchlist.checkdbssearchlink_0:rerunsearch/ED12066CDCF64031PQ/None?site=ibss&t:ac=RecentSearches) |
| S10 | [S5 OR S6 OR S8 OR S9](https://search.proquest.com/recentsearches.recentsearchtabview.recentsearchesgridview.scrolledrecentsearchlist.checkdbssearchlink:rerunsearch/B820244BC5AD45A4PQ/None?site=ibss&t:ac=RecentSearches) | [49,724](https://search.proquest.com/recentsearches.recentsearchtabview.recentsearchesgridview.scrolledrecentsearchlist.checkdbssearchlink_0:rerunsearch/B820244BC5AD45A4PQ/None?site=ibss&t:ac=RecentSearches) |
| S9 | [MAINSUBJECT.EXACT("Class size") OR MAINSUBJECT.EXACT("School class size") OR MAINSUBJECT.EXACT("Inclusive education") OR MAINSUBJECT.EXACT("Mainstreaming") OR MAINSUBJECT.EXACT("Teacher-student ratio")](https://search.proquest.com/recentsearches.recentsearchtabview.recentsearchesgridview.scrolledrecentsearchlist.checkdbssearchlink:rerunsearch/D4B6B23D5FA641CBPQ/None?site=ibss&t:ac=RecentSearches) | [1,370](https://search.proquest.com/recentsearches.recentsearchtabview.recentsearchesgridview.scrolledrecentsearchlist.checkdbssearchlink_0:rerunsearch/D4B6B23D5FA641CBPQ/None?site=ibss&t:ac=RecentSearches) |
| S8 | [(group* OR class*) N/5 (size*)](https://search.proquest.com/recentsearches.recentsearchtabview.recentsearchesgridview.scrolledrecentsearchlist.checkdbssearchlink:rerunsearch/5C3A6C40B8F849B2PQ/None?site=ibss&t:ac=RecentSearches) | [30,060](https://search.proquest.com/recentsearches.recentsearchtabview.recentsearchesgridview.scrolledrecentsearchlist.checkdbssearchlink_0:rerunsearch/5C3A6C40B8F849B2PQ/None?site=ibss&t:ac=RecentSearches) |
| S6 | [AB(group* OR class*) AND AB(size* OR ratio*)](https://search.proquest.com/recentsearches.recentsearchtabview.recentsearchesgridview.scrolledrecentsearchlist.checkdbssearchlink:rerunsearch/52ABE603359B4C8CPQ/None?site=ibss&t:ac=RecentSearches) | [23,132](https://search.proquest.com/recentsearches.recentsearchtabview.recentsearchesgridview.scrolledrecentsearchlist.checkdbssearchlink_0:rerunsearch/52ABE603359B4C8CPQ/None?site=ibss&t:ac=RecentSearches) |
| S5 | [TI(group* OR class*) AND TI(size* OR ratio*)](https://search.proquest.com/recentsearches.recentsearchtabview.recentsearchesgridview.scrolledrecentsearchlist.checkdbssearchlink:rerunsearch/86D8A9E52BE740C9PQ/None?site=ibss&t:ac=RecentSearches) | [934](https://search.proquest.com/recentsearches.recentsearchtabview.recentsearchesgridview.scrolledrecentsearchlist.checkdbssearchlink_0:rerunsearch/86D8A9E52BE740C9PQ/None?site=ibss&t:ac=RecentSearches) |
| S4 | [S1 OR S2 OR S3](https://search.proquest.com/recentsearches.recentsearchtabview.recentsearchesgridview.scrolledrecentsearchlist.checkdbssearchlink:rerunsearch/72C291D18E34C20PQ/None?site=ibss&t:ac=RecentSearches) | [19,865](https://search.proquest.com/recentsearches.recentsearchtabview.recentsearchesgridview.scrolledrecentsearchlist.checkdbssearchlink_0:rerunsearch/72C291D18E34C20PQ/None?site=ibss&t:ac=RecentSearches) |
| S3 | [MAINSUBJECT.EXACT("Special needs children") OR MAINSUBJECT.EXACT("Special education")](https://search.proquest.com/recentsearches.recentsearchtabview.recentsearchesgridview.scrolledrecentsearchlist.checkdbssearchlink:rerunsearch/649DAFD3BB134295PQ/None?site=ibss&t:ac=RecentSearches) | [1,362](https://search.proquest.com/recentsearches.recentsearchtabview.recentsearchesgridview.scrolledrecentsearchlist.checkdbssearchlink_0:rerunsearch/649DAFD3BB134295PQ/None?site=ibss&t:ac=RecentSearches) |
| S2 | [AB(special*) AND AB(need* OR education OR child* OR student* OR pupil*)](https://search.proquest.com/recentsearches.recentsearchtabview.recentsearchesgridview.scrolledrecentsearchlist.checkdbssearchlink:rerunsearch/79DF51DFF4594793PQ/None?site=ibss&t:ac=RecentSearches) | [18,544](https://search.proquest.com/recentsearches.recentsearchtabview.recentsearchesgridview.scrolledrecentsearchlist.checkdbssearchlink_0:rerunsearch/79DF51DFF4594793PQ/None?site=ibss&t:ac=RecentSearches) |
| S1 | [TI(special*) AND TI(need* OR education OR child* OR student* OR pupil*)](https://search.proquest.com/recentsearches.recentsearchtabview.recentsearchesgridview.scrolledrecentsearchlist.checkdbssearchlink:rerunsearch/4C12C30EB3584EF6PQ/None?site=ibss&t:ac=RecentSearches) | [1,427](https://search.proquest.com/recentsearches.recentsearchtabview.recentsearchesgridview.scrolledrecentsearchlist.checkdbssearchlink_0:rerunsearch/4C12C30EB3584EF6PQ/None?site=ibss&t:ac=RecentSearches) |

**Sociological Abstracts** (1952–2021). Search performed 23/04/2021. Search performed through the ProQuest advanced search interface.

| [Set](https://search.proquest.com/recentsearches.recentsearchtabview.recentsearchesgridview:toggellistorder?site=sociologicalabstracts&t:ac=RecentSearches) | **Search** | **Results** |
| --- | --- | --- |
| S16 | [S4 AND S8 AND S15](https://search.proquest.com/recentsearches.recentsearchtabview.recentsearchesgridview.scrolledrecentsearchlist.checkdbssearchlink:rerunsearch/C2A5094C0E964721PQ/None?site=sociologicalabstracts&t:ac=RecentSearches) | [476](https://search.proquest.com/recentsearches.recentsearchtabview.recentsearchesgridview.scrolledrecentsearchlist.checkdbssearchlink_0:rerunsearch/C2A5094C0E964721PQ/None?site=sociologicalabstracts&t:ac=RecentSearches) |
| S15 | [S9 OR S10 OR S11 OR S12 OR S13 OR S14](https://search.proquest.com/recentsearches.recentsearchtabview.recentsearchesgridview.scrolledrecentsearchlist.checkdbssearchlink:rerunsearch/2276BB5B62D1427EPQ/None?site=sociologicalabstracts&t:ac=RecentSearches) | [925,249](https://search.proquest.com/recentsearches.recentsearchtabview.recentsearchesgridview.scrolledrecentsearchlist.checkdbssearchlink_0:rerunsearch/2276BB5B62D1427EPQ/None?site=sociologicalabstracts&t:ac=RecentSearches) |
| S14 | [MAINSUBJECT.EXACT("Qualitative studies") OR MAINSUBJECT.EXACT("Qualitative research") OR MAINSUBJECT.EXACT(“Qualitative analysis") MAINSUBJECT.EXACT("Ethnography") OR MAINSUBJECT.EXACT("case studies") OR MAINSUBJECT.EXACT("interviews") OR MAINSUBJECT.EXACT("focus groups")](https://search.proquest.com/recentsearches.recentsearchtabview.recentsearchesgridview.scrolledrecentsearchlist.checkdbssearchlink:rerunsearch/50636AE455B14F0CPQ/None?site=sociologicalabstracts&t:ac=RecentSearches) | [33,502](https://search.proquest.com/recentsearches.recentsearchtabview.recentsearchesgridview.scrolledrecentsearchlist.checkdbssearchlink_0:rerunsearch/50636AE455B14F0CPQ/None?site=sociologicalabstracts&t:ac=RecentSearches) |
| S13 | [AB(qualitative* OR ethnograp* OR “case stud*” OR evaluation* OR “focus group*” OR interview* OR “mixed method*” OR observation*)](https://search.proquest.com/recentsearches.recentsearchtabview.recentsearchesgridview.scrolledrecentsearchlist.checkdbssearchlink:rerunsearch/EA9D53345E8644E1PQ/None?site=sociologicalabstracts&t:ac=RecentSearches) | [358,688](https://search.proquest.com/recentsearches.recentsearchtabview.recentsearchesgridview.scrolledrecentsearchlist.checkdbssearchlink_0:rerunsearch/EA9D53345E8644E1PQ/None?site=sociologicalabstracts&t:ac=RecentSearches) |
| S12 | [TI(qualitative* OR ethnograp* OR “case stud*” OR evaluation* OR “focus group*” OR interview* OR “mixed method*” OR observation*)](https://search.proquest.com/recentsearches.recentsearchtabview.recentsearchesgridview.scrolledrecentsearchlist.checkdbssearchlink:rerunsearch/65C19817E75F4BE6PQ/None?site=sociologicalabstracts&t:ac=RecentSearches) | [68,856](https://search.proquest.com/recentsearches.recentsearchtabview.recentsearchesgridview.scrolledrecentsearchlist.checkdbssearchlink_0:rerunsearch/65C19817E75F4BE6PQ/None?site=sociologicalabstracts&t:ac=RecentSearches) |
| S11 | [MAINSUBJECT.EXACT("Effectiveness") OR MAINSUBJECT.EXACT("Effects") OR MAINSUBJECT.EXACT("Experiments") OR MAINSUBJECT.EXACT("Intervention")](https://search.proquest.com/recentsearches.recentsearchtabview.recentsearchesgridview.scrolledrecentsearchlist.checkdbssearchlink:rerunsearch/AF9B0292ACB74C5EPQ/None?site=sociologicalabstracts&t:ac=RecentSearches) | [47,575](https://search.proquest.com/recentsearches.recentsearchtabview.recentsearchesgridview.scrolledrecentsearchlist.checkdbssearchlink_0:rerunsearch/AF9B0292ACB74C5EPQ/None?site=sociologicalabstracts&t:ac=RecentSearches) |
| S10 | [AB(effect* OR trial* OR experiment* OR "control group*" OR random* OR impact* OR compar* OR difference*)](https://search.proquest.com/recentsearches.recentsearchtabview.recentsearchesgridview.scrolledrecentsearchlist.checkdbssearchlink:rerunsearch/67AC8B2610D74D1FPQ/None?site=sociologicalabstracts&t:ac=RecentSearches) | [638,119](https://search.proquest.com/recentsearches.recentsearchtabview.recentsearchesgridview.scrolledrecentsearchlist.checkdbssearchlink_0:rerunsearch/67AC8B2610D74D1FPQ/None?site=sociologicalabstracts&t:ac=RecentSearches) |
| S9 | [TI(effect* OR trial* OR experiment* OR "control group*" OR random* OR impact* OR compar* OR difference*)](https://search.proquest.com/recentsearches.recentsearchtabview.recentsearchesgridview.scrolledrecentsearchlist.checkdbssearchlink:rerunsearch/CEBF2DE7C9954349PQ/None?site=sociologicalabstracts&t:ac=RecentSearches) | [152,091](https://search.proquest.com/recentsearches.recentsearchtabview.recentsearchesgridview.scrolledrecentsearchlist.checkdbssearchlink_0:rerunsearch/CEBF2DE7C9954349PQ/None?site=sociologicalabstracts&t:ac=RecentSearches) |
| S8 | [S5 OR S6 OR S7](https://search.proquest.com/recentsearches.recentsearchtabview.recentsearchesgridview.scrolledrecentsearchlist.checkdbssearchlink:rerunsearch/BB2C0799FDF2494FPQ/None?site=sociologicalabstracts&t:ac=RecentSearches) | [30,985](https://search.proquest.com/recentsearches.recentsearchtabview.recentsearchesgridview.scrolledrecentsearchlist.checkdbssearchlink_0:rerunsearch/BB2C0799FDF2494FPQ/None?site=sociologicalabstracts&t:ac=RecentSearches) |
| S7 | [(group* OR class*) N/5 (size*)](https://search.proquest.com/recentsearches.recentsearchtabview.recentsearchesgridview.scrolledrecentsearchlist.checkdbssearchlink:rerunsearch/2330C1274E7E438EPQ/None?site=sociologicalabstracts&t:ac=RecentSearches) | [14,758](https://search.proquest.com/recentsearches.recentsearchtabview.recentsearchesgridview.scrolledrecentsearchlist.checkdbssearchlink_0:rerunsearch/2330C1274E7E438EPQ/None?site=sociologicalabstracts&t:ac=RecentSearches) |
| S6 | [AB(group* OR class*) AND AB(size* OR ratio*)](https://search.proquest.com/recentsearches.recentsearchtabview.recentsearchesgridview.scrolledrecentsearchlist.checkdbssearchlink:rerunsearch/DDE5265D45C4503PQ/None?site=sociologicalabstracts&t:ac=RecentSearches) | [19,505](https://search.proquest.com/recentsearches.recentsearchtabview.recentsearchesgridview.scrolledrecentsearchlist.checkdbssearchlink_0:rerunsearch/DDE5265D45C4503PQ/None?site=sociologicalabstracts&t:ac=RecentSearches) |
| S5 | [TI(group* OR class*) AND TI(size* OR ratio*)](https://search.proquest.com/recentsearches.recentsearchtabview.recentsearchesgridview.scrolledrecentsearchlist.checkdbssearchlink:rerunsearch/82CC3431BDDA4F8EPQ/None?site=sociologicalabstracts&t:ac=RecentSearches) | [415](https://search.proquest.com/recentsearches.recentsearchtabview.recentsearchesgridview.scrolledrecentsearchlist.checkdbssearchlink_0:rerunsearch/82CC3431BDDA4F8EPQ/None?site=sociologicalabstracts&t:ac=RecentSearches) |
| S4 | [S1 OR S2 OR S3](https://search.proquest.com/recentsearches.recentsearchtabview.recentsearchesgridview.scrolledrecentsearchlist.checkdbssearchlink:rerunsearch/4B0CB78A33E44D0FPQ/None?site=sociologicalabstracts&t:ac=RecentSearches) | [26,458](https://search.proquest.com/recentsearches.recentsearchtabview.recentsearchesgridview.scrolledrecentsearchlist.checkdbssearchlink_0:rerunsearch/4B0CB78A33E44D0FPQ/None?site=sociologicalabstracts&t:ac=RecentSearches) |
| S3 | [MAINSUBJECT.EXACT(”Special Education”) OR MAINSUBJECT.EXACT(“Developmental Disabilities”)](https://search.proquest.com/recentsearches.recentsearchtabview.recentsearchesgridview.scrolledrecentsearchlist.checkdbssearchlink:rerunsearch/EB218BE759A4573PQ/None?site=sociologicalabstracts&t:ac=RecentSearches) | [2,128](https://search.proquest.com/recentsearches.recentsearchtabview.recentsearchesgridview.scrolledrecentsearchlist.checkdbssearchlink_0:rerunsearch/EB218BE759A4573PQ/None?site=sociologicalabstracts&t:ac=RecentSearches) |
| S2 | [AB(special*) AND AB(need* OR education OR child* OR student* OR pupil*)](https://search.proquest.com/recentsearches.recentsearchtabview.recentsearchesgridview.scrolledrecentsearchlist.checkdbssearchlink:rerunsearch/ACCE524A8A104FB7PQ/None?site=sociologicalabstracts&t:ac=RecentSearches) | [24,940](https://search.proquest.com/recentsearches.recentsearchtabview.recentsearchesgridview.scrolledrecentsearchlist.checkdbssearchlink_0:rerunsearch/ACCE524A8A104FB7PQ/None?site=sociologicalabstracts&t:ac=RecentSearches) |
| S1 | [TI(special*) AND TI(need* OR education OR child* OR student* OR pupil*)](https://search.proquest.com/recentsearches.recentsearchtabview.recentsearchesgridview.scrolledrecentsearchlist.checkdbssearchlink:rerunsearch/868A3F348FF54925PQ/None?site=sociologicalabstracts&t:ac=RecentSearches) | [1,691](https://search.proquest.com/recentsearches.recentsearchtabview.recentsearchesgridview.scrolledrecentsearchlist.checkdbssearchlink_0:rerunsearch/868A3F348FF54925PQ/None?site=sociologicalabstracts&t:ac=RecentSearches) |

**Web of Science** (**Clarivate**, **Science Citation Index Expanded**, 1900-2021; **Social Sciences Citation Index**, 1956-2021). Search performed 23/04/2021. Search performed through the Web of Science advanced search interface.

| **Search** | **Results** | **Search Terms** |
| --- | --- | --- |
| # 10 | [4,910](http://apps.webofknowledge.com/summary.do?product=WOS&doc=1&qid=20&SID=E6kWWICwmpBbdLTJgNj&search_mode=CombineSearches&update_back2search_link_param=yes) | #9 AND #6 AND #3  *Indexes=SCI-EXPANDED, SSCI Timespan=All years* |
| # 9 | [21,785,072](http://apps.webofknowledge.com/summary.do?product=WOS&doc=1&qid=19&SID=E6kWWICwmpBbdLTJgNj&search_mode=CombineSearches&update_back2search_link_param=yes) | #8 OR #7  *Indexes=SCI-EXPANDED, SSCI Timespan=All years* |
| # 8 | [3,972,826](http://apps.webofknowledge.com/summary.do?product=WOS&doc=1&qid=18&SID=E6kWWICwmpBbdLTJgNj&search_mode=AdvancedSearch&update_back2search_link_param=yes) | TI=(qualitative* OR ethnograp* OR “case stud*” OR evaluation* OR “focus group*” OR interview* OR “mixed method*” OR observation*) OR AB=(qualitative* OR ethnograp* OR “case stud*” OR evaluation* OR “focus group*” OR interview* OR “mixed method*” OR observation*)  *Indexes=SCI-EXPANDED, SSCI Timespan=All years* |
| # 7 | [20,228,327](http://apps.webofknowledge.com/summary.do?product=WOS&doc=1&qid=17&SID=E6kWWICwmpBbdLTJgNj&search_mode=AdvancedSearch&update_back2search_link_param=yes) | TI=(effect* OR trial* OR experiment* OR "control group*" OR random* OR impact* OR compar* OR difference*) OR AB=(effect* OR trial* OR experiment* OR "control group*" OR random* OR impact* OR compar* OR difference*)  *Indexes=SCI-EXPANDED, SSCI Timespan=All years* |
| # 6 | [884,456](http://apps.webofknowledge.com/summary.do?product=WOS&doc=1&qid=16&SID=E6kWWICwmpBbdLTJgNj&search_mode=CombineSearches&update_back2search_link_param=yes) | #5 OR #4  *Indexes=SCI-EXPANDED, SSCI Timespan=All years* |
| # 5 | [881,969](http://apps.webofknowledge.com/summary.do?product=WOS&doc=1&qid=11&SID=E6kWWICwmpBbdLTJgNj&search_mode=AdvancedSearch&update_back2search_link_param=yes) | AB=(group* OR class*) AND AB=(size* OR ratio*)  *Indexes=SCI-EXPANDED, SSCI Timespan=All years* |
| # 4 | [7,388](http://apps.webofknowledge.com/summary.do?product=WOS&doc=1&qid=10&SID=E6kWWICwmpBbdLTJgNj&search_mode=AdvancedSearch&update_back2search_link_param=yes) | TI=(group* OR class*) AND TI=(size* OR ratio*)  *Indexes=SCI-EXPANDED, SSCI Timespan=All years* |
| # 3 | [163,256](http://apps.webofknowledge.com/summary.do?product=WOS&doc=1&qid=9&SID=E6kWWICwmpBbdLTJgNj&search_mode=CombineSearches&update_back2search_link_param=yes) | #2 OR #1  *Indexes=SCI-EXPANDED, SSCI Timespan=All years* |
| # 2 | [157,605](http://apps.webofknowledge.com/summary.do?product=WOS&doc=1&qid=8&SID=E6kWWICwmpBbdLTJgNj&search_mode=AdvancedSearch&update_back2search_link_param=yes) | AB=(special*) AND AB=(need* OR education OR child* OR student* OR pupil*)  *Indexes=SCI-EXPANDED, SSCI Timespan=All years* |
| # 1 | [10,476](http://apps.webofknowledge.com/summary.do?product=WOS&doc=1&qid=7&SID=E6kWWICwmpBbdLTJgNj&search_mode=AdvancedSearch&update_back2search_link_param=yes) | TI=(special*) AND TI=(need* OR education OR child* OR student* OR pupil*)  *Indexes=SCI-EXPANDED, SSCI Timespan=All years* |

## 4 Hand-searches in key journals

Hand-searches were carried out from January to May 2022.

| **Journal** | **Year** | **Volume (number)** | **References screened** |
| --- | --- | --- | --- |
| **Exceptional Children** | 2015 | 82 (1) | 8 |
|  | 2015 | 81 (4) | 6 |
|  | 2015 | 81 (3) | 6 |
|  | 2015 | 81 (2) | 7 |
|  | 2016 | 81 (1) | 7 |
|  | 2016 | 82 (4) | 6 |
|  | 2016 | 82 (3) | 6 |
|  | 2016 | 82 (2) | 5 |
|  | 2017 | 84 (1) | 6 |
|  | 2017 | 83 (4) | 6 |
|  | 2017 | 83 (3) | 6 |
|  | 2017 | 83 (2) | 6 |
|  | 2018 | 85 (1) | 6 |
|  | 2018 | 84 (4) | 6 |
|  | 2018 | 84 (3) | 6 |
|  | 2018 | 84 (2) | 6 |
|  | 2019 | 86 (1) | 6 |
|  | 2019 | 85 (4) | 5 |
|  | 2019 | 85 (3) | 7 |
|  | 2019 | 85 (2) | 9 |
|  | 2020 | 87 (1) | 4 |
|  | 2020 | 86 (4) | 6 |
|  | 2020 | 86 (3) | 6 |
|  | 2020 | 86 (2) | 6 |
|  | 2021 | 88 (1) | 6 |
|  | 2021 | 87 (4) | 6 |
|  | 2021 | 87 (3) | 6 |
|  | 2021 | 87 (2) | 6 |
|  | 2022 | 88 (2) | 6 |
|  |  |  |  |
| **Learning Disability Quarterly** | 2015 | 38 (4) | 5 |
|  | 2015 | 38 (3) | 4 |
|  | 2015 | 38 (2) | 4 |
|  | 2015 | 38 (1) | 5 |
|  | 2016 | 39 (4) | 5 |
|  | 2016 | 39 (3) | 6 |
|  | 2016 | 39 (2) | 5 |
|  | 2016 | 39 (1) | 6 |
|  | 2017 | 40 (4) | 4 |
|  | 2017 | 40 (3) | 6 |
|  | 2017 | 40 (2) | 6 |
|  | 2017 | 40 (1) | 5 |
|  | 2018 | 41 (4) | 5 |
|  | 2018 | 41 (3) | 5 |
|  | 2018 | 41 (2) | 6 |
|  | 2018 | 41 (1) | 5 |
|  | 2019 | 42 (4) | 5 |
|  | 2019 | 42 (3) | 4 |
|  | 2019 | 42 (2) | 5 |
|  | 2019 | 42 (1) | 4 |
|  | 2020 | 43 (4) | 5 |
|  | 2020 | 43 (3) | 5 |
|  | 2020 | 43 (2) | 5 |
|  | 2020 | 43 (1) | 4 |
|  | 2021 | 44 (4) | 6 |
|  | 2021 | 44 (3) | 7 |
|  | 2021 | 44 (2) | 5 |
|  | 2021 | 44 (1) | 5 |
|  | 2022 | 45 (1) | 5 |
|  |  |  |  |
| **Behavioral Disorders** | 2015 | 40 (2) | 4 |
|  | 2015 | 40 (3) | 4 |
|  | 2015 | 40 (4) | 3 |
|  | 2015 | 41 (1) | 5 |
|  | 2016 | 41 (2) | 4 |
|  | 2016 | 41 (3) | 3 |
|  | 2016 | 41 (4) | 6 |
|  | 2016 | 42 (1) | 3 |
|  | 2017 | 42 (2) | 4 |
|  | 2017 | 42 (3) | 5 |
|  | 2017 | 42 (4) | 5 |
|  | 2017 | 43 (1) | 7 |
|  | 2018 | 43 (2) | 5 |
|  | 2018 | 43 (3) | 6 |
|  | 2018 | 43 (4) | 5 |
|  | 2018 | 44 (1) | 4 |
|  | 2019 | 44 (2) | 6 |
|  | 2019 | 44 (3) | 4 |
|  | 2019 | 44 (4) | 5 |
|  | 2019 | 45 (1) | 5 |
|  | 2020 | 45 (2) | 4 |
|  | 2020 | 45 (3) | 4 |
|  | 2020 | 45 (4) | 4 |
|  | 2020 | 46 (1) | 4 |
|  | 2021 | 46 (2) | 4 |
|  | 2021 | 46 (3) | 5 |
|  | 2021 | 46 (4) | 6 |
|  | 2021 | 47 (2) | 5 |
|  | 2022 | 47 (2) | 5 |
|  |  |  |  |
| **Journal of Autism & Developmental Disorders** | 2015 | 45 (1) | 27 |
|  | 2015 | 45 (2) | 25 |
|  | 2015 | 46 (3) | 24 |
|  | 2015 | 45 (4) | 21 |
|  | 2015 | 45 (5) | 34 |
|  | 2015 | 45 (6) | 34 |
|  | 2015 | 45 (7) | 35 |
|  | 2015 | 45 (8) | 35 |
|  | 2015 | 45 (9) | 35 |
|  | 2015 | 45 (10) | 32 |
|  | 2015 | 45 (11) | 34 |
|  | 2015 | 45 (12) | 40 |
|  | 2016 | 46 (1) | 35 |
|  | 2016 | 46 (2) | 33 |
|  | 2016 | 46 (3) | 36 |
|  | 2016 | 46 (4) | 36 |
|  | 2016 | 46 (5) | 35 |
|  | 2016 | 46 (6) | 35 |
|  | 2016 | 46 (7) | 31 |
|  | 2016 | 46 (8) | 25 |
|  | 2016 | 46 (9) | 30 |
|  | 2016 | 46 (10) | 18 |
|  | 2016 | 46 (11) | 19 |
|  | 2016 | 46 (12) | 21 |
|  | 2017 | 47 (1) | 20 |
|  | 2017 | 47 (2) | 29 |
|  | 2017 | 47 (3) | 32 |
|  | 2017 | 47 (4) | 30 |
|  | 2017 | 47 (5) | 29 |
|  | 2017 | 47 (6) | 32 |
|  | 2017 | 47 (7) | 31 |
|  | 2017 | 47 (8) | 31 |
|  | 2017 | 47 (9) | 30 |
|  | 2017 | 47 (10) | 30 |
|  | 2017 | 47 (11) | 30 |
|  | 2017 | 47 (12) | 31 |
|  | 2018 | 48 (1) | 30 |
|  | 2018 | 48 (2) | 29 |
|  | 2018 | 48 (3) | 30 |
|  | 2018 | 48 (4) | 39 |
|  | 2018 | 48 (5) | 37 |
|  | 2018 | 48 (6) | 36 |
|  | 2018 | 48 (7) | 30 |
|  | 2018 | 48 (8) | 30 |
|  | 2018 | 48 (9) | 34 |
|  | 2018 | 48 (10) | 32 |
|  | 2018 | 48 (11) | 30 |
|  | 2018 | 48 (12) | 30 |
|  | 2019 | 49 (1) | 41 |
|  | 2019 | 49 (2) | 30 |
|  | 2019 | 49 (3) | 41 |
|  | 2019 | 49 (4) | 35 |
|  | 2019 | 49 (5) | 35 |
|  | 2019 | 49 (6) | 39 |
|  | 2019 | 49 (7) | 35 |
|  | 2019 | 49 (8) | 35 |
|  | 2019 | 49 (9) | 36 |
|  | 2019 | 49 (10) | 38 |
|  | 2019 | 49 (11) | 33 |
|  | 2019 | 49 (12) | 33 |
|  | 2020 | 50 (1) | 33 |
|  | 2020 | 50 (2) | 33 |
|  | 2020 | 50 (3) | 33 |
|  | 2020 | 50 (4) | 32 |
|  | 2020 | 50 (5) | 35 |
|  | 2020 | 50 (6) | 34 |
|  | 2020 | 50 (7) | 34 |
|  | 2020 | 50 (8) | 33 |
|  | 2020 | 50 (9) | 33 |
|  | 2020 | 50 (10) | 32 |
|  | 2020 | 50 (11) | 33 |
|  | 2020 | 50 (12) | 32 |
|  | 2021 | 51 (1) | 33 |
|  | 2021 | 51 (2) | 33 |
|  | 2021 | 51 (3) | 24 |
|  | 2021 | 51 (4) | 32 |
|  | 2021 | 51 (5) | 34 |
|  | 2021 | 51 (6) | 33 |
|  | 2021 | 51 (7) | 35 |
|  | 2021 | 51 (8) | 35 |
|  | 2021 | 51 (9) | 51 |
|  | 2021 | 51 (10) | 33 |
|  | 2021 | 51 (11) | 39 |
|  | 2021 | 51 (12) | 40 |
|  | 2022 | 52 (1) | 40 |
|  |  |  |  |
| **Remedial and Special Education** | 2015 | 36 (1) | 12 |
|  | 2015 | 36 (2) | 10 |
|  | 2015 | 36 (3) | 5 |
|  | 2015 | 36 (4) | 5 |
|  | 2015 | 36 (5) | 5 |
|  | 2015 | 36 (6) | 5 |
|  | 2016 | 37 (1) | 5 |
|  | 2016 | 37 (2) | 5 |
|  | 2016 | 37 (3) | 4 |
|  | 2016 | 37 (4) | 6 |
|  | 2016 | 37 (5) | 5 |
|  | 2016 | 37 (6) | 5 |
|  | 2017 | 38 (1) | 5 |
|  | 2017 | 38 (2) | 5 |
|  | 2017 | 38 (3) | 5 |
|  | 2017 | 38 (4) | 5 |
|  | 2017 | 38 (5) | 5 |
|  | 2017 | 38 (6) | 5 |
|  | 2018 | 39 (1) | 5 |
|  | 2018 | 39 (2) | 5 |
|  | 2018 | 39 (3) | 5 |
|  | 2018 | 39 (4) | 5 |
|  | 2018 | 39 (5) | 4 |
|  | 2018 | 39 (6) | 6 |
|  | 2019 | 40 (1) | 5 |
|  | 2019 | 40 (2) | 7 |
|  | 2019 | 40 (3) | 5 |
|  | 2019 | 40 (4) | 4 |
|  | 2019 | 40 (5) | 7 |
|  | 2019 | 40 (6) | 5 |
|  | 2020 | 41 (1) | 5 |
|  | 2020 | 41 (2) | 5 |
|  | 2020 | 41 (3) | 6 |
|  | 2020 | 41 (4) | 6 |
|  | 2020 | 41 (5) | 5 |
|  | 2020 | 41 (6) | 5 |
|  | 2021 | 42 (1) | 6 |
|  | 2021 | 42 (2) | 5 |
|  | 2021 | 42 (3) | 5 |
|  | 2021 | 42 (4) | 6 |
|  | 2021 | 42 (5) | 6 |
|  | 2021 | 42 (6) | 5 |
|  |  |  |  |
| **British Journal of Special Education** | 2015 | 42 (1) | 7 |
|  | 2015 | 42 (2) | 5 |
|  | 2015 | 42 (3) | 5 |
|  | 2015 | 42 (4) | 5 |
|  | 2016 | 43 (1) | 5 |
|  | 2016 | 43 (2) | 5 |
|  | 2016 | 43 (3) | 5 |
|  | 2016 | 43 (4) | 6 |
|  | 2017 | 44 (1) | 5 |
|  | 2017 | 44 (2) | 5 |
|  | 2017 | 44 (3) | 6 |
|  | 2017 | 44 (4) | 6 |
|  | 2018 | 45 (1) | 5 |
|  | 2018 | 45 (2) | 5 |
|  | 2018 | 45 (3) | 7 |
|  | 2018 | 45 (4) | 5 |
|  | 2019 | 46 (1) | 5 |
|  | 2019 | 46 (2) | 5 |
|  | 2019 | 46 (3) | 5 |
|  | 2019 | 46 (4) | 5 |
|  | 2020 | 47 (1) | 5 |
|  | 2020 | 47 (2) | 5 |
|  | 2020 | 47 (3) | 5 |
|  | 2020 | 47 (4) | 5 |
|  | 2021 | 48 (1) | 5 |
|  | 2021 | 48 (2) | 5 |
|  | 2021 | 48 (3) | 5 |
|  | 2021 | 48 (4) | 5 |
|  |  |  |  |
| **Learning Disabilities Research & Practice** | 2015 | 30 (1) | 4 |
|  | 2015 | 30 (2) | 4 |
|  | 2015 | 30 (3) | 4 |
|  | 2015 | 30 (4) | 5 |
|  | 2016 | 31 (1) | 4 |
|  | 2016 | 31 (2) | 5 |
|  | 2016 | 31 (3) | 4 |
|  | 2016 | 31 (4) | 4 |
|  | 2017 | 32 (1) | 6 |
|  | 2017 | 32 (2) | 5 |
|  | 2017 | 32 (3) | 6 |
|  | 2017 | 32 (4) | 6 |
|  | 2018 | 33 (1) | 4 |
|  | 2018 | 33 (2) | 6 |
|  | 2018 | 33 (3) | 4 |
|  | 2018 | 33 (4) | 6 |
|  | 2019 | 34 (1) | 5 |
|  | 2019 | 34 (2) | 4 |
|  | 2019 | 34 (3) | 4 |
|  | 2019 | 34 (4) | 4 |
|  | 2020 | 35 (1) | 5 |
|  | 2020 | 35 (2) | 5 |
|  | 2020 | 35 (3) | 5 |
|  | 2020 | 35 (4) | 4 |
|  | 2021 | 36 (1) | 5 |
|  | 2021 | 36 (2) | 7 |
|  | 2021 | 36 (3) | 8 |
|  | 2021 | 36 (4) | 6 |
|  |  |  |  |
| **Journal of Intellectual Disability Research** | 2015 | 59 (1) | 8 |
|  | 2015 | 59 (2) | 10 |
|  | 2015 | 59 (3) | 9 |
|  | 2015 | 59 (4) | 9 |
|  | 2015 | 59 (5) | 9 |
|  | 2015 | 59 (6) | 9 |
|  | 2015 | 59 (7) | 8 |
|  | 2015 | 59 (8) | 1 |
|  | 2015 | 59 (9) | 7 |
|  | 2015 | 59 (10) | 9 |
|  | 2015 | 59 (11) | 7 |
|  | 2015 | 59 (12) | 8 |
|  | 2016 | 60 (1) | 9 |
|  | 2016 | 60 (2) | 9 |
|  | 2016 | 60 (3) | 10 |
|  | 2016 | 60 (4) | 9 |
|  | 2016 | 60 (5) | 10 |
|  | 2016 | 60 (6) | 9 |
|  | 2016 | 60 (7) | 4 |
|  | 2016 | 60 (8) | 9 |
|  | 2016 | 60 (9) | 11 |
|  | 2016 | 60 (10) | 9 |
|  | 2016 | 60 (11) | 9 |
|  | 2016 | 60 (12) | 9 |
|  | 2017 | 61 (1) | 8 |
|  | 2017 | 61 (2) | 7 |
|  | 2017 | 61 (3) | 9 |
|  | 2017 | 61 (4) | 7 |
|  | 2017 | 61 (5) | 9 |
|  | 2017 | 61 (6) | 10 |
|  | 2017 | 61 (7) | 9 |
|  | 2017 | 61 (8) | 8 |
|  | 2017 | 61 (9) | 9 |
|  | 2017 | 61 (10) | 8 |
|  | 2017 | 61 (11) | 7 |
|  | 2017 | 61 (12) | 10 |
|  | 2018 | 62 (1) | 8 |
|  | 2018 | 62 (2) | 8 |
|  | 2018 | 62 (3) | 9 |
|  | 2018 | 62 (4) | 8 |
|  | 2018 | 62 (5) | 9 |
|  | 2018 | 62 (6) | 10 |
|  | 2018 | 62 (7) | 6 |
|  | 2018 | 62 (8) | 8 |
|  | 2018 | 62 (9) | 7 |
|  | 2018 | 62 (10) | 8 |
|  | 2018 | 62 (11) | 6 |
|  | 2018 | 62 (12) | 11 |
|  | 2019 | 63 (1) | 8 |
|  | 2019 | 63 (2) | 8 |
|  | 2019 | 63 (3) | 8 |
|  | 2019 | 63 (4) | 9 |
|  | 2019 | 63 (5) | 15 |
|  | 2019 | 63 (6) | 15 |
|  | 2019 | 63 (7) | 12 |
|  | 2019 | 63 (8) | 10 |
|  | 2019 | 63 (9) | 9 |
|  | 2019 | 63 (10) | 8 |
|  | 2019 | 63 (11) | 8 |
|  | 2019 | 63 (12) | 9 |
|  | 2020 | 64 (1) | 8 |
|  | 2020 | 64 (2) | 8 |
|  | 2020 | 64 (3) | 7 |
|  | 2020 | 64 (4) | 6 |
|  | 2020 | 64 (5) | 6 |
|  | 2020 | 64 (6) | 7 |
|  | 2020 | 64 (7) | 8 |
|  | 2020 | 64 (8) | 8 |
|  | 2020 | 64 (9) | 7 |
|  | 2020 | 64 (10) | 7 |
|  | 2020 | 64 (11) | 7 |
|  | 2020 | 64 (12) | 8 |
|  | 2021 | 65 (1) | 8 |
|  | 2021 | 65 (2) | 8 |
|  | 2021 | 65 (3) | 7 |
|  | 2021 | 65 (4) | 7 |
|  | 2021 | 65 (5) | 7 |
|  | 2021 | 65 (6) | 7 |
|  | 2021 | 65 (7) | 7 |
|  | 2021 | 65 (8) | 4 |
|  | 2021 | 65 (9) | 7 |
|  | 2021 | 65 (10) | 6 |
|  | 2021 | 65 (11) | 7 |
|  | 2021 | 65 (12) | 7 |
|  | 2022 | 66 (1-2) | 14 |
|  | 2022 | 66 (3) | 7 |
|  |  |  |  |
| **International Journal of Disability, Development & Education** | 2015 | 62 (6) | 9 |
|  | 2015 | 62 (5) | 5 |
|  | 2015 | 62 (4) | 7 |
|  | 2015 | 62 (3) | 6 |
|  | 2015 | 62 (2) | 6 |
|  | 2015 | 62 (1) | 7 |
|  | 2016 | 63 (6) | 5 |
|  | 2016 | 63 (5) | 5 |
|  | 2016 | 64 (4) | 5 |
|  | 2016 | 64 (3) | 8 |
|  | 2016 | 65 (2) | 6 |
|  | 2016 | 65 (1) | 7 |
|  | 2017 | 64 (6) | 5 |
|  | 2017 | 64 (5) | 6 |
|  | 2017 | 64 (4) | 7 |
|  | 2017 | 64 (3) | 7 |
|  | 2017 | 64 (2) | 7 |
|  | 2017 | 64 (1) | 7 |
|  | 2018 | 65 (6) | 6 |
|  | 2018 | 65 (5) | 6 |
|  | 2018 | 65 (4) | 7 |
|  | 2018 | 65 (3) | 6 |
|  | 2018 | 65 (2) | 7 |
|  | 2018 | 65 (1) | 8 |
|  | 2019 | 66 (6) | 6 |
|  | 2019 | 66 (5) | 6 |
|  | 2019 | 66 (4) | 7 |
|  | 2019 | 66 (3) | 6 |
|  | 2019 | 66 (2) | 9 |
|  | 2019 | 66 (1) | 7 |
|  | 2020 | 67 (6) | 5 |
|  | 2020 | 67 (5) | 7 |
|  | 2020 | 67 (4) | 7 |
|  | 2020 | 67 (3) | 7 |
|  | 2020 | 67 (2) | 8 |
|  | 2020 | 67 (1) | 8 |
|  | 2021 | 68 (6) | 7 |
|  | 2021 | 68 (5) | 8 |
|  | 2021 | 68 (4) | 9 |
|  | 2021 | 68 (3) | 11 |
|  | 2021 | 68 (2) | 10 |
|  |  |  |  |
| **Journal of Speech, Language, and Hearing Research** | 2015 | 58 (1) | 10 |
|  | 2015 | 58 (2) | 27 |
|  | 2015 | 58 (3) | 37 |
|  | 2015 | 58 (4) | 22 |
|  | 2015 | 58 (5) | 14 |
|  | 2015 | 58 (6) | 10 |
|  | 2016 | 59 (1) | 10 |
|  | 2016 | 59 (2) | 15 |
|  | 2016 | 59 (3) | 15 |
|  | 2016 | 59 (4) | 19 |
|  | 2016 | 59 (5) | 21 |
|  | 2016 | 59 (6) | 27 |
|  | 2017 | 60 (1) | 22 |
|  | 2017 | 60 (2) | 13 |
|  | 2017 | 60 (3) | 17 |
|  | 2017 | 60 (4) | 22 |
|  | 2017 | 60 (5) | 19 |
|  | 2017 | 60 (6) | 14 |
|  | 2017 | 60 (6S) | 10 |
|  | 2017 | 60 (7) | 18 |
|  | 2017 | 60 (8) | 15 |
|  | 2017 | 60 (9) | 23 |
|  | 2017 | 60 (10) | 15 |
|  | 2017 | 60 (11) | 22 |
|  | 2017 | 60 (12) | 22 |
|  | 2018 | 61 (1) | 10 |
|  | 2018 | 61 (2) | 12 |
|  | 2018 | 61 (3) | 19 |
|  | 2018 | 61 (4) | 18 |
|  | 2018 | 61 (5) | 18 |
|  | 2018 | 61 (6) | 11 |
|  | 2018 | 61 (7) | 20 |
|  | 2018 | 61 (8) | 17 |
|  | 2018 | 61 (9) | 17 |
|  | 2018 | 61 (10) | 11 |
|  | 2018 | 61 (11) | 8 |
|  | 2018 | 61 (12) | 15 |
|  | 2019 | 62 (1) | 12 |
|  | 2019 | 62 (2) | 16 |
|  | 2019 | 62 (3) | 17 |
|  | 2019 | 62 (4) | 17 |
|  | 2019 | 62 (4S) | 9 |
|  | 2019 | 62 (5) | 21 |
|  | 2019 | 62 (6) | 27 |
|  | 2019 | 62 (7) | 28 |
|  | 2019 | 62 (8) | 20 |
|  | 2019 | 62 (8S) | 10 |
|  | 2019 | 62 (9) | 31 |
|  | 2019 | 62 (10) | 18 |
|  | 2019 | 62 (11) | 14 |
|  | 2019 | 62 (12) | 24 |
|  | 2020 | 63 (1) | 22 |
|  | 2020 | 63 (2) | 19 |
|  | 2020 | 63 (3) | 16 |
|  | 2020 | 63 (4) | 25 |
|  | 2020 | 63 (5) | 19 |
|  | 2020 | 63 (6) | 24 |
|  | 2020 | 63 (7) | 29 |
|  | 2020 | 63 (8) | 22 |
|  | 2020 | 63 (9) | 23 |
|  | 2020 | 63 (10) | 15 |
|  | 2020 | 63 (11) | 21 |
|  | 2020 | 63 (12) | 25 |
|  | 2021 | 64 (1) | 17 |
|  | 2021 | 64 (2) | 21 |
|  | 2021 | 64 (3) | 24 |
|  | 2021 | 64 (4) | 22 |
|  | 2021 | 64 (5) | 18 |
|  | 2021 | 64 (6) | 17 |
|  | 2021 | 64 (6S) | 19 |
|  | 2021 | 64 (7) | 26 |
|  | 2021 | 64 (8) | 21 |
|  | 2021 | 64 (9) | 20 |
|  | 2021 | 64 (10) | 20 |
|  | 2021 | 64 (11) | 24 |
|  | 2021 | 64 (12) | 27 |
|  | 2022 | 65 (1) | 22 |
|  | 2022 | 65 (2) | 23 |

| **European**  **Educational**  **Research Journal** | 2015 | 14 (1) | 5 |
| --- | --- | --- | --- |
|  | 2015 | 14 (2) | 4 |
|  | 2015 | 14 (3-4) | 10 |
|  | 2015 | 14 (5) | 4 |
|  | 2015 | 14 (6) | 5 |
|  | 2016 | 15 (1) | 8 |
|  | 2016 | 15 (2) | 5 |
|  | 2016 | 15 (3) | 6 |
|  | 2016 | 15 (4) | 8 |
|  | 2016 | 15 (5) | 5 |
|  | 2016 | 15 (6) | 6 |
|  | 2017 | 16 (1) | 6 |
|  | 2017 | 16 (2-3) | 14 |
|  | 2017 | 16 (4) | 8 |
|  | 2017 | 16 (5) | 9 |
|  | 2017 | 16 (6) | 11 |
|  | 2018 | 17 (1) | 10 |
|  | 2018 | 17 (2) | 6 |
|  | 2018 | 17 (3) | 7 |
|  | 2018 | 17 (4) | 7 |
|  | 2018 | 17 (5) | 6 |
|  | 2018 | 17 (6) | 8 |
|  | 2019 | 18 (1) | 7 |
|  | 2019 | 18 (2) | 7 |
|  | 2019 | 18 (3) | 7 |
|  | 2019 | 18 (4) | 6 |
|  | 2019 | 18 (5) | 8 |
|  | 2019 | 18 (6) | 6 |
|  | 2020 | 19 (1) | 5 |
|  | 2020 | 19 (2) | 5 |
|  | 2020 | 19 (3) | 4 |
|  | 2020 | 19 (4) | 5 |
|  | 2020 | 19 (5) | 6 |
|  | 2020 | 19 (6) | 6 |
|  | 2021 | 20 (1) | 5 |
|  | 2021 | 20 (2) | 7 |
|  | 2021 | 20 (3) | 8 |
|  | 2021 | 20 (4) | 7 |
|  | 2021 | 20 (5) | 7 |
|  | 2021 | 20 (6) | 11 |
|  | 2022 | 21 (1) | 10 |
|  | 2022 | 21 (2) | 10 |
|  | 2022 | 21 (3) | 9 |

## 5 Searches for unpublished literature

**EBSCO OPEN Dissertations.** Search performed 23/04/2021.

| **Search** | **Search Terms** | **Results** |
| --- | --- | --- |
| S10 | S3 AND S6 AND S9 | 139 |
| S9 | S7 OR S8 | 203,049 |
| S8 | TI (qualitative* OR ethnograp* OR “case stud*” OR evaluation* OR “focus group*” OR interview* OR “mixed method*” OR observation*) | 52,241 |
| S7 | TI (effect* OR trial* OR experiment* OR "control group*" OR random* OR impact* OR compar* OR difference*) | 156,452 |
| S6 | S4 OR S5 | 33,834 |
| S5 | AB (group* OR class*) AND AB (size* OR ratio*) | 33,717 |
| S4 | TI (group* OR class*) AND TI (size* OR ratio*) | 221 |
| S3 | S1 OR S2 | 15,954 |
| S2 | AB (special*) AND AB (need* OR education OR child* OR student* OR pupil*) | 14,989 |
| S1 | TI (special*) AND TI (need* OR education OR child* OR student* OR pupil*) | 2,071 |

**Google Scholar:** <https://scholar.google.com/>. Search performed 29/03/2022.

Standard searches were performed using the following combinations of terms covering the population and the intervention:

- "special needs students" AND "class size" AND "special education"
- "special needs pupils" AND "class size" AND "special education"
- "special needs children" AND "class size" AND "special education"
- "students with special educational needs" AND "class size" AND "special education"
- "children with special educational needs" AND "class size" AND "special education"
- "pupils with special educational needs" AND "class size" AND "special education"
- "children with disabilities" AND "class size" AND "special education"
- "students with disabilities" AND "class size" AND "special education"
- "pupils with disabilities" AND "class size" AND "special education"
- "exceptional children" AND "class size" AND "special education"
- "special class" AND "class size"
- "special school" AND "class size"
- "self-contained" AND "class size"
- "resource room" AND "class size"
- "segregated placement" AND "class size"
- "special needs students" AND "small class*" AND "special education"
- "students with disabilities" AND "small class*" AND "special education"
- "self-contained" AND "small class*"

The searches yielded 1.900 references which were screened against the inclusion/exclusion criteria.

**Social Science Research Network:** <https://www.ssrn.com/index.cfm/en/>. Search performed 29/03/2022.

Advanced searches were performed with the following selections: Education, Cognitive Science, Sociology, Public health, and ALL SSRN Networks, using the following combinations of terms covering the population and the intervention:

- "special needs students" "class size" "special education"
- special needs students
- class size
- special needs students class size special education
- class size special education
- small class special education
- "class size"
- "class size" "special education"
- "small class" "special education"

The searches yielded 117 references which were screened against the inclusion/exclusion criteria.

**OECD iLibrary:** <https://www.oecd-ilibrary.org/>. Search performed 06/04/2022.

Advanced searches were performed using the following combinations of terms covering the population and the intervention:

- "special needs students" AND "class size" AND "special education"
- "special needs pupils" AND "class size" AND "special education"
- "special needs children" AND "class size" AND "special education"
- "students with special educational needs" AND "class size" AND "special education"
- "children with special educational needs" AND "class size" AND "special education"
- "special needs students" AND "small class*" AND "special education"
- "students with disabilities" AND "small class*" AND "special education"
- "self-contained" AND "small class*"

The searches yielded 711 references which were screened against the inclusion/exclusion criteria.

**National Bureau of Economic Research (NBER):** [http://www.nber.org](http://www.nber.org/). Search performed 06/04/2022.

Standard searches were performed using the following combinations of terms covering the population and the intervention:

- "special needs students", "class size", "special education"
- "special needs pupils" "class size" "special education"
- "special needs children" "class size" "special education"
- "students with disabilities" "class size" "special education"
- "resource room" "class size"
- "segregated placement" "class size"
- "self-contained" "class size"

The searches yielded 700 references which were screened against the inclusion/exclusion criteria.

**American Educational Research Association (AERA):** <https://www.aera.net/>. Search performed 06/04/2022.

Standard searches were performed using the following combinations of terms covering the population and the intervention:

- "special needs students" "class size" "special education"
- "special needs pupils" "class size" "special education"
- "resource room" "class size"
- "self-contained" "class size"
- "children with disabilities" "class size"
- "small classes" "class size" "Teacher Student"

The searches yielded 435 references which were screened against the inclusion/exclusion criteria.

**Social Care Online:** <https://www.scie-socialcareonline.org.uk/>. Search performed 06/04/2022.

Standard searches were performed using the following combinations of terms covering the population and the intervention:

- "special needs students" "class size" "special education"
- special education class size
- special education small class
- "children with disabilities" class size special education
- "students with disabilities" class size special education
- "students with disabilities" small class special education
- "segregated placement" class size
- "special class" class size
- special class

The searches yielded 222 references which were screened against the inclusion/exclusion criteria.

**Best Evidence Encyclopaedia:** <http://www.bestevidence.org/>. Search performed 06/04/2022.

Standard searches were performed using the following combinations of terms covering the population and the intervention:

- special needs students "class size" "special education"
- special needs students class size special education
- special needs students class size
- special needs students
- class size
- special education

The searches yielded 27 references which were screened against the inclusion/exclusion criteria.

**Forskning.ku:** <https://forskning.ku.dk/soeg/>. Search performed 06/04/2022.

Standard searches were performed using the following combinations of terms covering the population and the intervention:

- class size special education
- class size special needs student
- small class* special education
- klassestørrelse* specialundervisning*
- klassestørrelse*
- elever med særlige behov klassestørrelse
- elever med særlige behov gruppestørrelse
- specialskole gruppestørrelse
- specialtilbud gruppestørrelse
- specialundervisning klassestørrelse
- specialundervisning klassestørrelse

The searches yielded 346 references which were screened against the inclusion/exclusion criteria.

**AAU Publications—Academic publications from the University of Aarhus:** <https://pure.au.dk/portal/da/organisations/8000/publications.html>. Search performed 06/04/2022.

Standard searches were performed using the following combinations of terms covering the population and the intervention:

- class size special education
- class size special needs student
- small class* special education
- klassestørrelse* specialundervisning*
- klassestørrelse*
- "children with disabilities" "class size"
- "children with disabilities" education
- "class size"
- "small class"
- "group size" "special education"
- "group size" education

The searches yielded 32 references which were screened against the inclusion/exclusion criteria.

**SwePub—Academic publications at Swedish universities:** <http://swepub.kb.se/>. Search performed 12/04/2022.

Advanced searches were performed using the following combinations of terms covering the population and the intervention:

- class size special education
- class size
- spesialundervisning klassestørrelse
- specialundervisning
- klasstorlek
- gruppstorlek
- elever med särskilda behov
- specialskola

The searches yielded 30 references which were screened against the inclusion/exclusion criteria.

**NORA—Norwegian Open Research Archives:** <http://nora.openaccess.no/>. Search performed 12/04/2022.

Standard searches were performed using the following combinations of terms covering the population and the intervention:

- class size special education
- small class* special education
- "special class" class size
- "children with disabilities" "class size"
- "class size"
- "small class"
- Special education

The searches yielded 134 references which were screened against the inclusion/exclusion criteria.

**DIVA—Swedish Digital Scientific Archives:** <http://www.diva-portal.org/smash/>. Search performed 12/04/2022.

Advanced searches were performed using the following combinations of terms covering the population and the intervention:

- "special needs students" AND "class size" AND "special education"
- "special needs students"
- "class size"
- "specialundervisning" AND klasstorlek
- specialundervisning
- klasstorlek
- gruppstorlek

The searches yielded 74 references which were screened against the inclusion/exclusion criteria.

**Skolporten—Swedish Dissertations:** <https://www.skolporten.se/forskning/>. Search performed 12/04/2022.

Searches were performed using the following combinations of terms covering the population and the intervention:

- "special needs students" AND "class size" AND "special education"
- special needs students
- class size
- klasstorlek
- gruppstorlek
- specialundervisning

The searches yielded 111 references which were screened against the inclusion/exclusion criteria.

**Campbell Journal of Systematic Reviews:** <https://campbellcollaboration.org/>. Search performed 12/04/2022.

Searches were performed using the following combinations of terms covering the population and the intervention:

- class size special education
- class size
- special education small class
- small class

The searches yielded 76 references which were screened against the inclusion/exclusion criteria.

**Cochrane Library:** <https://www.cochranelibrary.com/>. Search performed 12/04/2022.

Simple searches (title/abstract/keyword) were performed using the following combinations of terms covering the population and the intervention:

- "class size" "special education"
- "class size"
- "small class"
- "group size"
- "special education"
- "special class"
- special need students

The searches yielded 34 references which were screened against the inclusion/exclusion criteria.

**Centre for Reviews and Dissemination Databases:** <https://www.crd.york.ac.uk/CRDWeb/>. Search performed 13/04/2022.

Advanced searches were performed using the following combinations of terms covering the population and the intervention:

- class size AND special education
- class size AND special need students
- class size
- special education AND size
- special education
- segregated placement
- special need students

The searches yielded 78 references which were screened against the inclusion/exclusion criteria.

**EPPI-Centre Database of Education Research:** <https://eppi.ioe.ac.uk/webdatabases/Intro.aspx?ID=6>. Search performed 13/04/2022.

Searches were performed by free-text using the following combinations of terms covering the population and the intervention:

- "Class size" AND "Special education"
- "Small class" AND "Special education"
- "students with special needs" AND "Special education"
- "students with disabilities" AND "special education"
- "group size"
- "Small class"
- "special need students"
- "students with special needs"

The searches yielded 39 references which were screened against the inclusion/exclusion criteria.

**CORE—research outputs from international repositories:** <https://core.ac.uk/>. Search performed 13/04/2022.

Simple searches were performed using the following combinations of terms covering the population and the intervention:

- "Class size" AND "special education" AND "students with special needs"
- "students with disabilities" AND "small class*" AND "special education"
- "students with disabilities" AND "class size" AND "special education"
- special class AND "class size"
- "segregated placement" AND "class size"
- "elever med særlige behov" AND "klassestørrelse"
- "elever med særlige behov" AND "gruppestørrelse"
- klassestørrelse AND spesialundervisning
- klasstorlek AND specialundervisning
- gruppstorlek AND specialundervisning

The searches yielded 589 references which were screened against the inclusion/exclusion criteria.

**Google:** <https://www.google.com/>. Search performed 13/04/2022.

Simple searches were performed using the following combinations of terms covering the population and the intervention:

- "special needs students" AND "class size" AND "special education"
- "special needs children" AND "class size" AND "special education"
- "children with disabilities" AND "class size" AND "special education"
- students with disabilities AND "class size" AND "special education"
- "exceptional children" AND "class size" AND "special education"
- "special class" AND "class size"
- "special school" AND "class size"
- "self-contained" AND "class size"
- "resource room" AND "class size"
- "segregated placement " AND "class size"
- "special needs students" AND "small class*" AND "special education"
- "students with disabilities" AND "small class*" AND "special education"
- "self-contained" AND "small class*"

The searches yielded 1.300 references which were screened against the inclusion/exclusion criteria.

## 6 Citation-tracking and snowballing

We performed both forward and backward citation-tracking on the following reviews/research overviews: [McCrea, 1996](#REF-McCrea_x002c_-1996), [Ahearn, 1995](#REF-Ahearn_x002c_-1995), and [Zarghami, 2004](#REF-Zarghami_x002c_-2004).

In addition, we performed citation-tracking on the included references: [Forness, 1985](#STD-Forness_x002c_-1985), [Gottlieb, 1997](#STD-Gottlieb_x002c_-1997), [Huang, 2020](#STD-Huang_x002c_-2020), [Keith, 1993a](#STD-Keith_x002c_-1993a), [Metzner, 1926](#STD-Metzner_x002c_-1926), and [Prunty, 2012](#STD-Prunty_x002c_-2012). It was not possible to perform citation-tracking on [MAGI Educational Services, Inc., 1995](#STD-MAGI-Educational-Services_x002c_-In), since it did not contain a list of references.

A total of 499 references were found through citation-tracking and screened following the inclusion/exclusion criteria. Citation-tracking was performed between January and May 2022.
